# Performance augmentation of spherical solar distiller using dual-axis sun tracking system with corrugated absorber and phase change material

**DOI:** 10.1038/s41598-026-55272-8

**Published:** 2026-06-04

**Authors:** M. Ibrahim, M. M. Younes, Z. M. Omara, Fadl A. Essa

**Affiliations:** Mechanical Engineering Department, Faculty of Engineering, Kafrelsheikh University, Kafrelsheikh, Egypt

**Keywords:** Solar desalination enhancement, Spherical solar still, Thermal energy storage, Enhanced evaporation surface, Heat transfer augmentation, Solar energy utilization, Water scarcity solution, Energy science and technology, Engineering, Environmental sciences, Nanoscience and technology

## Abstract

Freshwater scarcity is a growing global challenge, particularly in arid regions where sustainable solutions are urgently required. Solar distillation is a promising technology; however, its low productivity limits large-scale implementation. In this study, a novel integrated modified spherical solar still (MSPSS) is proposed, combining vertical absorber configuration, dual-axis solar tracking, optimized corrugated geometry, and silver nanoparticle-enhanced phase change material (PCM-Ag) to overcome the limitations of previously reported designs. The proposed system introduces a synergistic integration of optical, thermal, and geometric enhancements, enabling superior performance compared to conventional and previously developed spherical solar stills. Experimental results demonstrate a significant increase in freshwater productivity, reaching a maximum of 13,300 mL/m^2^ day, corresponding to a 343% improvement over the conventional solar still (CSS). The individual contributions of the modifications include vertical absorber (62%), dual-axis tracking (65%), corrugated geometry (55%), and PCM-Ag integration (52%). In addition to productivity enhancement, the system improves thermal management by reducing temperature fluctuations and extending effective operating periods. Economic analysis reveals a reduction in freshwater production cost from $0.024/L for CSS to $0.01/L for the optimized configuration. These findings confirm that the proposed integrated approach represents a substantial advancement over existing spherical solar still systems, offering an efficient and economically viable solution for decentralized freshwater production in water-scarce regions.

## Introduction

### Background and motivation

The global water crisis has intensified significantly in recent decades, with approximately 2.2 billion people lacking access to safely managed drinking water services according to recent assessments^1^. Population growth, industrial expansion, agricultural demands, and climate change have collectively exacerbated water stress across numerous regions worldwide. Traditional water treatment infrastructure requires substantial capital investment, continuous energy input, and technical expertise for operation and maintenance, making it economically prohibitive for many developing communities, particularly those in remote or rural areas^2^.

Solar distillation presents a compelling alternative technology that harnesses abundant solar radiation to purify contaminated water through natural evaporation and condensation processes^3,4^. This passive water treatment method requires minimal maintenance, operates without external energy input, and can function effectively in off-grid locations where conventional treatment facilities are impractical. The fundamental principle involves heating saline or brackish water using solar energy, causing evaporation of pure water vapor, which subsequently condenses on cooler surfaces and is collected as fresh water^5,6^. Despite these advantages, conventional solar stills have historically suffered from low productivity rates, typically ranging from 2 to 5 L per square meter per day, which has limited their widespread adoption^7,8^.

The efficiency of solar distillation depends on multiple interrelated factors including solar radiation intensity, ambient temperature, wind speed, water depth, absorber characteristics, condensation surface area, and thermal properties of construction materials^9^. Researchers have explored numerous enhancement techniques to improve productivity, including material modifications, geometric optimizations, thermal energy storage integration, and active tracking systems^8,10^. However, most previous studies have focused on individual enhancement methods rather than comprehensive, integrated approaches that combine multiple strategies synergistically.

### Solar still configurations and advancement

Conventional solar stills typically employ simple basin-type designs with horizontal absorber plates covered by transparent glass or plastic enclosures^11^. While these systems demonstrate reliable operation and durability, their productivity remains constrained by limited evaporation surface area, suboptimal solar radiation absorption angles, and thermal losses. Single-slope stills represent the most common configuration due to their simplicity and low cost, yet they experience significant shading effects during morning and evening hours when the sun’s position creates unfavorable incident angles^12,13^.

Spherical solar stills have emerged as a promising alternative configuration that addresses several limitations of conventional designs^14,15^. The spherical geometry provides a larger condensation surface area relative to the base area, potentially increasing freshwater collection. Additionally, the curved glass cover receives solar radiation at more favorable angles throughout the day compared to flat surfaces, reducing reflection losses and improving energy absorption^16^. The spherical configuration also minimizes self-shading effects that plague conventional basin-type stills during off-peak hours.

Despite these advantages, spherical solar stills have received relatively limited research attention compared to traditional basin-type designs. Most existing studies on spherical stills have focused on passive configurations without active enhancement techniques. The integration of advanced features such as solar tracking systems, optimized absorber geometries, and thermal storage materials represents an underexplored area with significant potential for performance improvement.

### Solar tracking systems

Solar tracking systems have been extensively investigated for photovoltaic applications, where maintaining perpendicular panel orientation to incident sunlight can increase energy capture by 20–40% compared to fixed installations^17^. These systems employ mechanical mechanisms controlled by sensors or astronomical algorithms to follow the sun’s apparent motion across the sky. Two primary tracking configurations exist: single-axis systems that rotate along one dimension (typically east-west azimuth tracking), and dual-axis systems that adjust both azimuth and elevation angles to maintain optimal orientation throughout the day and across seasons^18,19^.

Single-axis trackers offer a compromise between performance improvement and system complexity, providing substantial gains over fixed systems while requiring relatively simple mechanical components and control algorithms. These trackers typically rotate along a north-south horizontal axis to follow the sun’s daily east-west trajectory, but cannot compensate for seasonal elevation changes. Consequently, their performance advantage varies significantly with latitude and season, with maximum benefits occurring during equinoxes when the sun’s path aligns closely with the tracker’s rotational plane^20^.

Dual-axis tracking systems provide superior performance by continuously adjusting both azimuthal and elevational angles to maintain perpendicular alignment with incoming solar radiation^21^. This comprehensive tracking capability ensures optimal energy capture regardless of time of day or season, making dual-axis systems particularly advantageous in regions with significant seasonal variation in solar elevation angles. Advanced dual-axis trackers can increase energy collection by 35–45% compared to fixed installations, with the greatest improvements occurring during winter months when solar elevation angles are lowest^22,23^.

Despite their proven effectiveness in photovoltaic applications, solar tracking systems have rarely been applied to solar distillation systems^24,25^. The few existing studies on tracked solar stills have primarily focused on basin-type configurations rather than spherical designs, and have not comprehensively evaluated the interaction between tracking systems and other enhancement techniques such as modified absorber geometries or thermal storage materials.

### Absorber surface enhancement

The absorber surface plays a crucial role in solar still performance by converting incident solar radiation into thermal energy that drives water evaporation^26,27^. Conventional flat absorbers provide a uniform heat transfer surface but offer limited evaporation area relative to the still’s footprint. Researchers have explored various absorber modifications including surface treatments (black paint, selective coatings), material alternatives (concrete, rubber, metals), and geometric enhancements to increase effective surface area^7,28^.

Corrugated or textured absorber surfaces represent a promising enhancement strategy that increases the effective evaporation area without expanding the still’s physical footprint^29^. The increased surface area promotes more intensive evaporation for a given water volume, potentially accelerating freshwater production. Additionally, corrugated geometries create turbulence in the water film, enhancing convective heat transfer and reducing thermal boundary layer thickness^30^. These combined effects can significantly improve evaporation rates compared to smooth flat absorbers.

Vertical absorber orientation presents another modification strategy that addresses water depth and heat distribution challenges^31^. Conventional horizontal absorbers require substantial water depth to cover the entire surface, increasing thermal mass and reducing temperature rise rates^32^. Vertical absorbers can be wetted with thin water films using capillary wicking materials, minimizing water inventory while maintaining complete surface coverage^33^. This configuration reduces thermal inertia, enabling faster temperature response to solar radiation variations and potentially extending productive operating hours.

### Phase change materials for thermal management

Phase change materials (PCMs) absorb or release substantial latent heat during phase transitions (typically solid-liquid), providing thermal energy storage capability without significant temperature change^34,35^. In solar still applications, PCMs can store excess thermal energy during peak radiation hours and release it during low-radiation periods, smoothing temperature fluctuations and extending productive operation into evening hours. Additionally, PCM integration can moderate glass cover temperatures, reducing heat losses while maintaining favorable temperature gradients for condensation^36^.

Nanoparticle enhancement of PCMs has emerged as an effective strategy for improving thermal performance characteristics^37^. Metallic nanoparticles such as silver, copper, or aluminum oxide dispersed within PCM matrices can significantly increase thermal conductivity, accelerating both charging and discharging rates^38,39^. This enhancement addresses a primary limitation of many organic PCMs, which exhibit low thermal conductivity that restricts heat transfer rates and limits practical effectiveness in dynamic thermal applications.

Silver nanoparticles offer particular advantages for PCM enhancement due to their exceptionally high thermal conductivity, antimicrobial properties, and chemical stability^3^. When properly dispersed at appropriate concentrations (typically 0.5–2% by weight), silver nanoparticles can increase PCM thermal conductivity by 20–50% without significantly affecting phase transition temperatures or latent heat capacity^3^. This performance enhancement can substantially improve thermal management effectiveness in solar distillation systems.

### Research objectives and novelty

This investigation addresses critical knowledge gaps regarding integrated enhancement strategies for spherical solar stills through comprehensive experimental evaluation of multiple combined modifications. The specific objectives include:

1. Assess the individual and combined effects of vertical absorber orientation, solar tracking, corrugated geometry, and PCM integration on spherical solar still productivity.
2. Characterize thermal behavior including water temperatures, glass cover temperatures, and temperature distributions under various enhancement configurations.
3. Evaluate the economic viability of enhanced systems through techno-economic analysis considering capital costs, productivity improvements, and freshwater production costs.
4. Provide practical design guidance for implementing advanced solar distillation systems in water-scarce regions.

The novelty of this research lies in the development of a fully integrated enhancement strategy for spherical solar stills, combining vertical corrugated absorber geometry, dual-axis solar tracking, and silver nanoparticle-enhanced phase change materials (PCM-Ag) within a single system. Unlike previously reported studies, which primarily focused on individual modifications or partial integrations, the present work provides a comprehensive and systematic evaluation of the combined effects of multiple enhancement techniques.

Furthermore, this study demonstrates a substantially higher performance improvement compared to previously reported spherical solar still systems, achieving up to 343% productivity enhancement. This advancement is attributed to the synergistic interaction between optimized solar tracking, enhanced heat transfer surfaces, and improved thermal energy storage. The integrated design not only enhances evaporation rates but also improves thermal stability and extends operational periods.

This work therefore represents a significant advancement in solar distillation technology, providing new insights into the design and optimization of high-performance spherical solar stills for practical and large-scale applications.

## Materials and methods

### Experimental setup

#### Conventional spherical solar still (CSS)

**Fig. 1:**
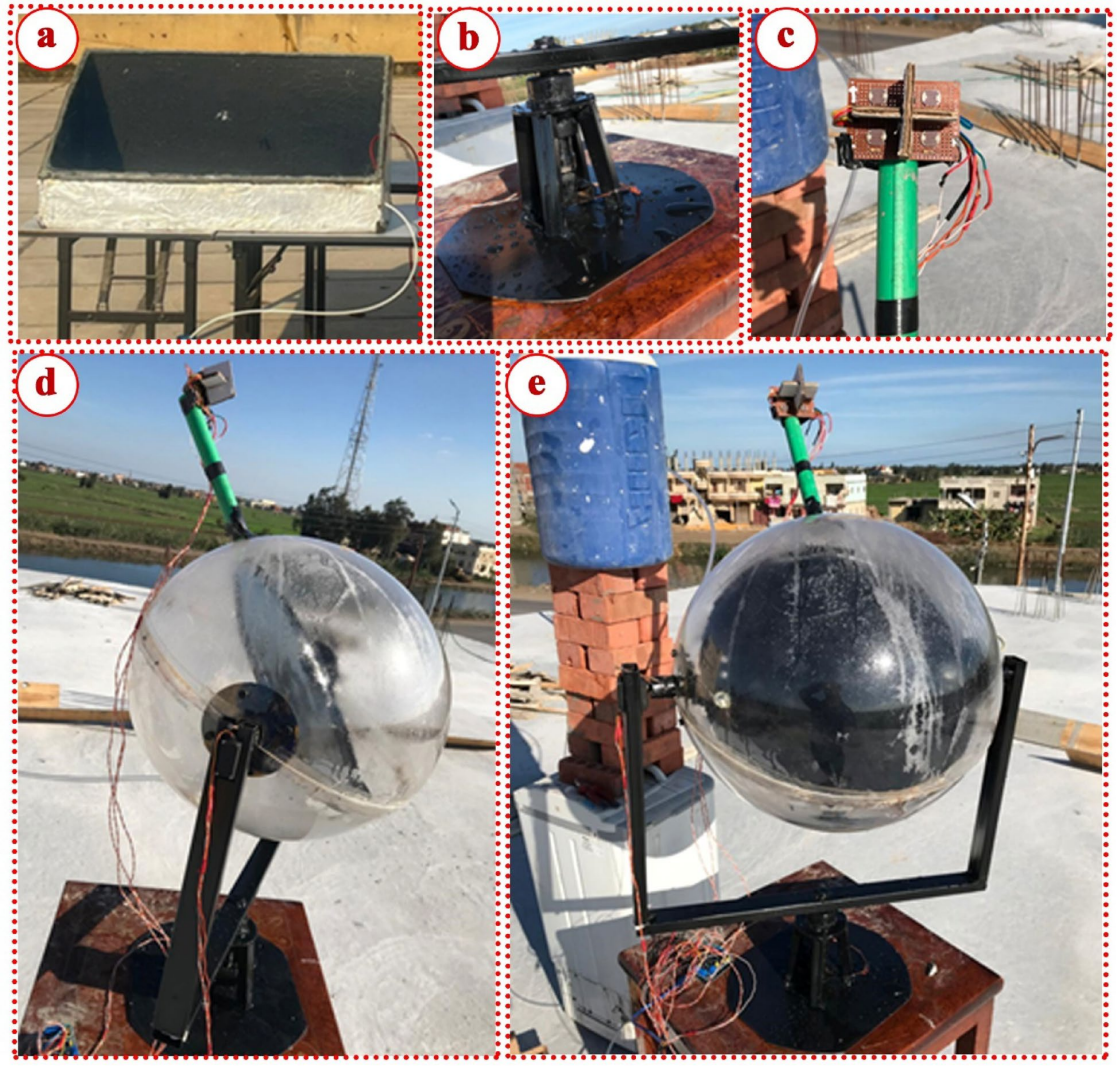
Photographs of the conventional and modified spherical solar stills: (**a**) CSS, (**b**) tracking system, (**c**) photosensor, (**d**) side view of MSPSS, and (**e**) front view of MSPSS.

**Fig. 2:**
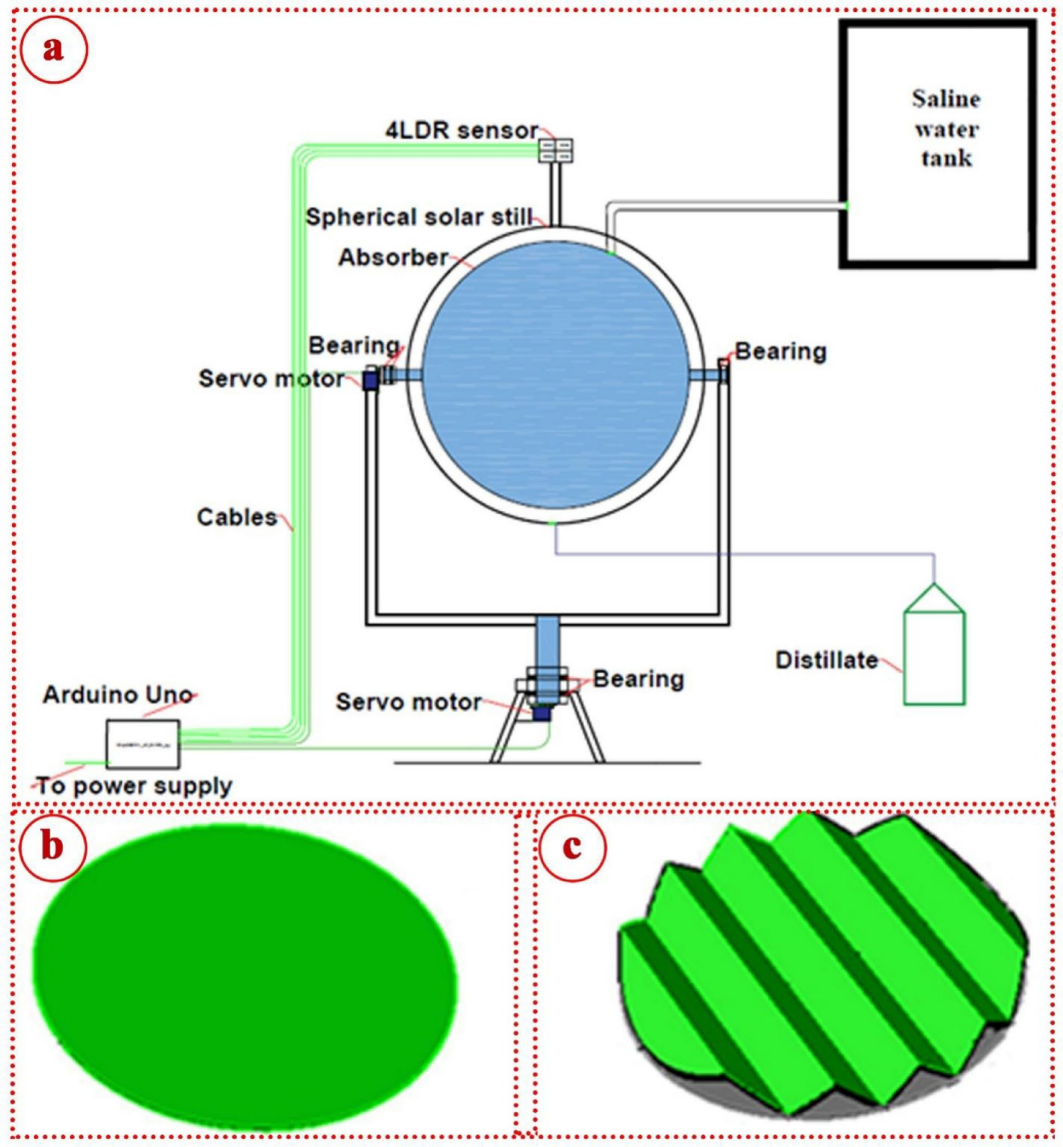
Schematic diagram of the modified spherical distiller: (**a**) MSPSS, (**b**) flat disc absorber, and (**c**) corrugated disc absorber.

Figure 1 illustrates the photographs of the conventional and modified spherical solar stills. While Fig. 2 presents schematic diagram of the modified spherical distiller with the various absorbers investigated. The baseline conventional spherical solar still (CSS) was constructed using a hemispherical transparent glass cover with 2 m^2^ surface area and 0.6 m^2^ base area. The still body was fabricated from galvanized steel sheet with thickness of 1.5 mm, coated with black paint to enhance solar radiation absorption. The basin was insulated using polyurethane foam with thermal conductivity of 0.026 W/m K and thickness of 50 mm to minimize bottom and side thermal losses. A horizontal flat absorber plate was positioned at the basin bottom, painted with matte black paint to maximize radiation absorption. The glass cover was sealed to the still body using silicone sealant to prevent vapor leakage, with a condensate collection channel integrated along the glass perimeter, draining to an external graduated cylinder for productivity measurement.

#### Modified spherical solar still (MSPSS) configurations

The modified spherical solar still incorporated several progressive enhancements evaluated systematically. The first modification (MSPSS-VFA) replaced the horizontal flat absorber with a vertical flat absorber positioned at the still center. The vertical absorber was wrapped with jute cloth material to maintain a thin water film through capillary action, reducing water inventory while maximizing wetted surface area. This configuration maintained the same spherical glass cover geometry as CSS.

The second modification (MSPSS-STVFA) added a single-axis solar tracking mechanism to the vertical flat absorber. This tracking system enabled east-west rotation following the sun’s daily azimuthal trajectory from sunrise to sunset. The tracking mechanism employed a servomotor-driven rotational stage mounted beneath the still, rotating the absorber assembly while maintaining the stationary glass cover.

The third modification (MSPSS-DTFA) implemented dual-axis solar tracking capability, enabling both azimuthal (east-west) and elevational (vertical angle) adjustment of the vertical flat absorber. This configuration utilized a more sophisticated mechanical system with two independent servomotors controlling rotation about perpendicular axes, enabling the absorber to maintain near-perpendicular orientation to incident solar radiation throughout the day and across seasons.

The fourth modification (MSPSS-DTCA) replaced the flat vertical absorber with a corrugated vertical absorber while maintaining dual-axis tracking capability. The corrugated absorber was fabricated by forming galvanized steel sheet into a sinusoidal profile with amplitude of 25 mm and wavelength of 50 mm. This corrugation pattern increased the effective surface area by approximately 86% compared to the equivalent flat absorber, while maintaining the same projected frontal area. The corrugated surface was wrapped with jute cloth identical to the flat absorber configuration.

The final modification (MSPSS-DTCA + PCM-Ag) integrated silver nanoparticle-enhanced phase change material behind the corrugated absorber while maintaining all previous enhancements. The PCM consisted of paraffin wax (melting point 52–54 °C) enhanced with 1% by weight silver nanoparticles (average diameter 40 nm). The PCM-Ag mixture was contained in a sealed aluminum enclosure with 20 mm thickness, positioned directly behind the corrugated absorber to provide thermal mass and temperature moderation during high-radiation periods.

### Solar tracking system design

#### Hardware components

**Fig. 3:**
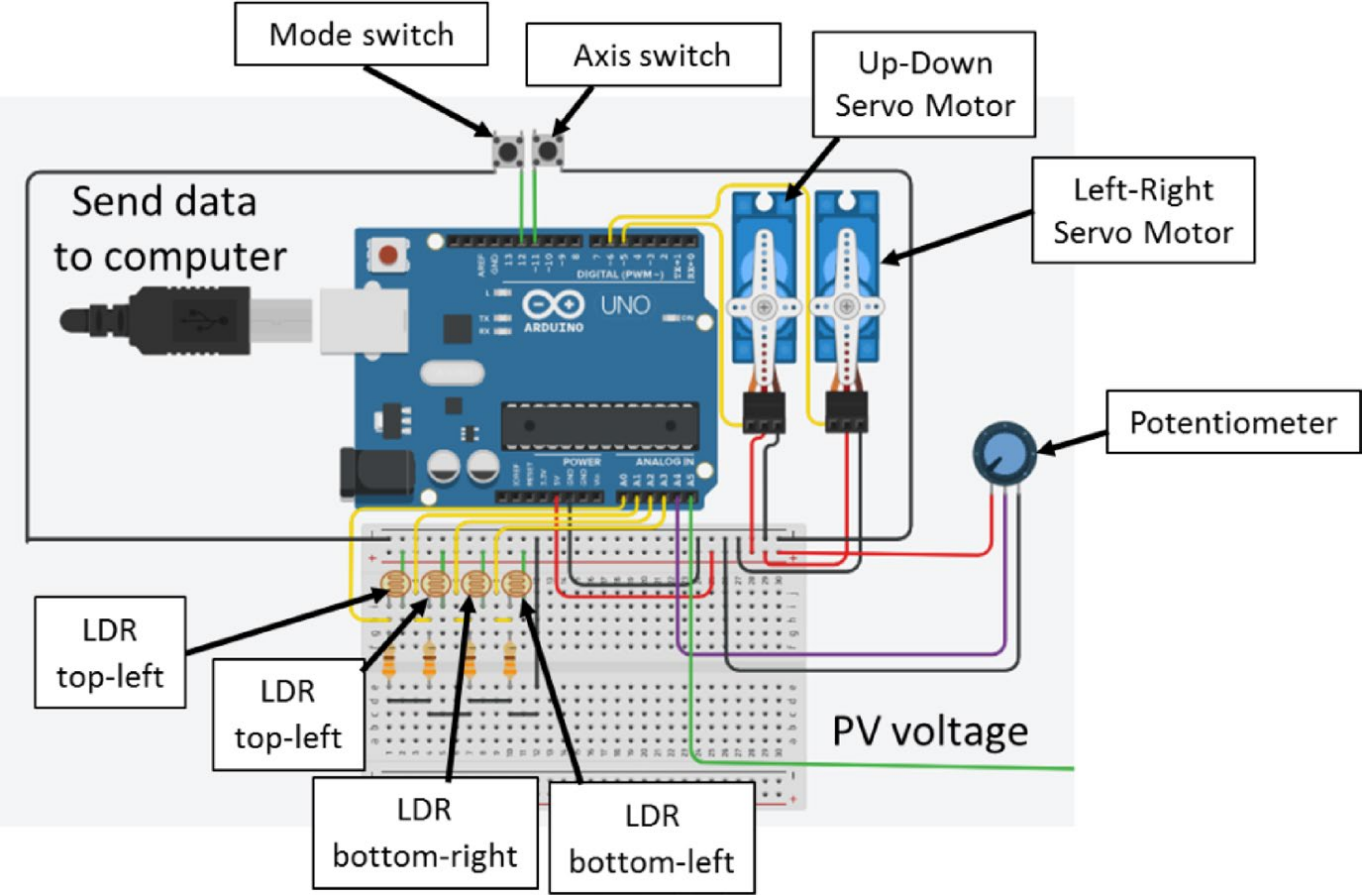
Electronic circuit of the solar tracker with manual and automatic modes.

Figure 3 illustrates the electronic circuit of the proposed test bench. The dual-axis solar tracking system was implemented using an Arduino Uno microcontroller (ATmega328P processor) as the central control unit. Sun position sensing utilized four Light-Dependent Resistors (LDRs) with dark resistance of 5 MΩ and light resistance of 100 Ω, arranged in a cruciform pattern to detect relative illumination from different directions. Each LDR was connected in a voltage divider circuit with a 330 Ω fixed resistor, producing analog voltage signals proportional to incident light intensity. These analog signals were fed to analog input pins A0-A3 of the Arduino for conversion to digital values using the built-in 10-bit ADC.

Mechanical actuation employed two MG996R servomotors, each capable of 180-degree rotation with torque of 11 kg cm at 6 V supply voltage. One servomotor (SM1) controlled azimuthal rotation about a vertical axis, while the second servomotor (SM2) controlled elevational angle adjustment about a horizontal axis. The servomotors received position commands via pulse-width modulation (PWM) signals from Arduino digital pins 5 and 6, enabling precise angular control with approximately 0.5-degree resolution. Table 1 presents the component of tracking control system.

**Table 1:** The component of tracking control system.

No.	Tools	Quantity
1	Resistor 330 ohm	4
2	LDR, 5 Mohm	4
3	Arduino UNO	1
4	Mini Solar Panel	1
5	MG996R servo motor	2
6	Rotary potentiometer (generic)	1
7	Pushbutton Switch, Pushbutton	2
8	Arduino IDE	1

The system included manual control capability using a rotary potentiometer (10 kΩ resistance) connected to analog input pin A4. Two pushbutton switches enabled mode selection (automatic vs. manual operation, pin 12) and servomotor selection for manual control (azimuth vs. elevation adjustment, pin 11). A small photovoltaic panel (6 V, 100 mA) powered the control electronics and provided voltage measurement for system monitoring via analog input pin A5.

#### Control algorithm

**Fig. 4:**
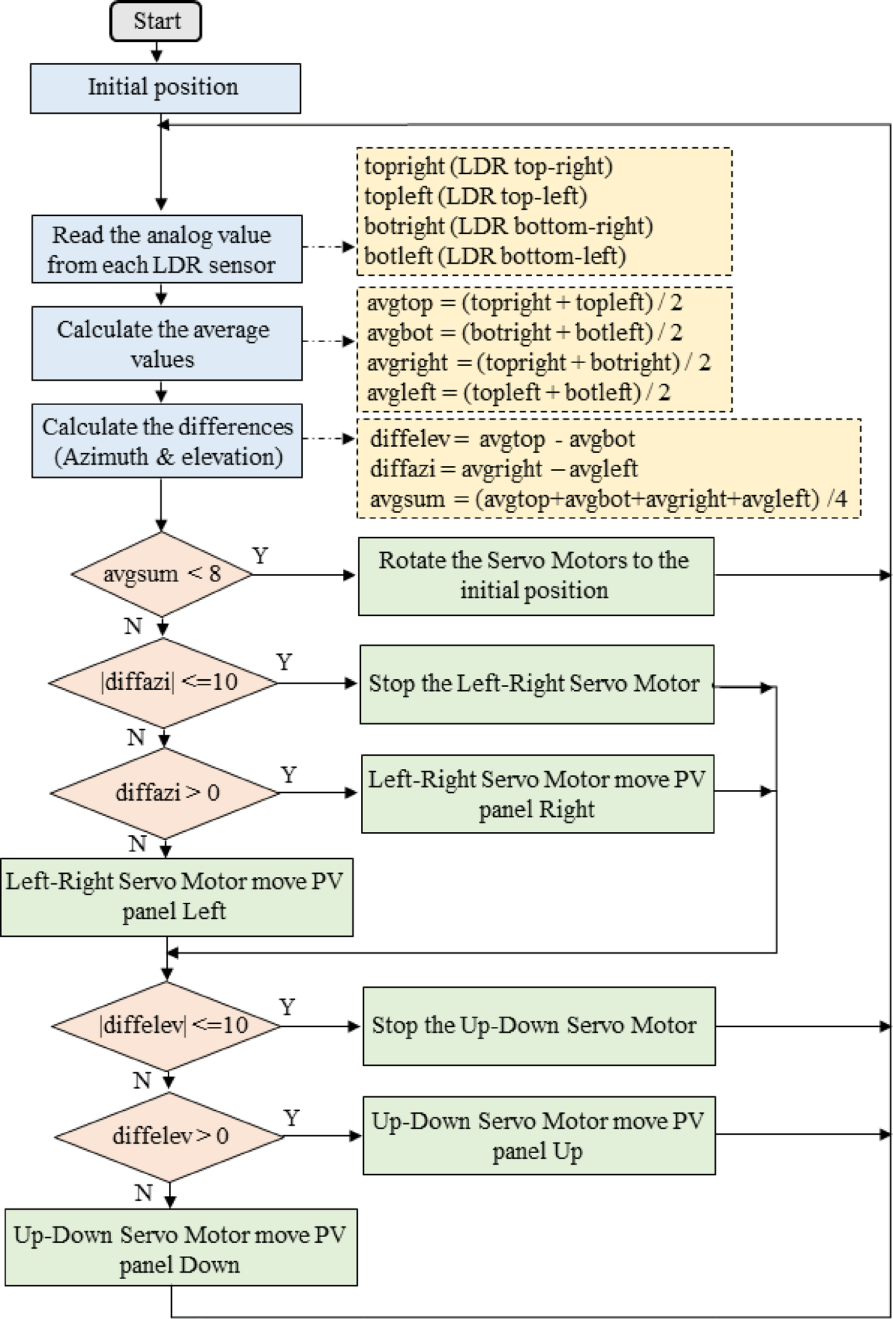
The algorithm for automatic mode of the solar tracker.

The automatic tracking algorithm, shown in Fig. 4, employed a differential light intensity approach to determine optimal tracking directions. For azimuthal tracking, the algorithm computed average values from the two left-side LDRs (sensors 1 and 3) and the two right-side LDRs (sensors 2 and 4). The difference between these averages indicated the direction of maximum illumination: positive difference signaled eastward (right) rotation requirement, while negative difference indicated westward (left) rotation. The servomotor adjusted position incrementally until the difference magnitude fell below a threshold value of 10 digital units (approximately 50 mV), indicating near-perpendicular alignment.

Elevational tracking followed an analogous approach, comparing average values from upper LDRs (sensors 1 and 2) versus lower LDRs (sensors 3 and 4). The elevation servomotor adjusted the absorber vertical angle based on this comparison, rotating until the difference magnitude fell within the threshold band. Both tracking axes updated continuously with a sampling period of 2 s, providing responsive adjustment to changing sun positions while avoiding oscillatory behavior.

The algorithm incorporated a night-time reset function to return the absorber to sunrise position during dark hours, conserving energy and ensuring optimal starting position for the following day. When the average illumination across all four LDRs fell below a threshold value of 8 digital units (indicating near-zero solar radiation), the system commanded both servomotors to predefined sunrise positions: 0 degrees azimuth (eastward orientation) and 30 degrees elevation. This reset procedure executed once when the darkness threshold was crossed, avoiding repetitive movements during extended dark periods.

Manual control mode enabled user override of automatic tracking for maintenance, testing, or demonstration purposes. In manual mode, the potentiometer position controlled servomotor angles directly, with the selection pushbutton determining which axis responded to potentiometer adjustment. This dual-mode capability provided operational flexibility while maintaining autonomous operation as the primary mode.

### Phase change material preparation

The PCM–Ag composite was synthesized using a two-step method involving nanoparticle dispersion followed by thermal stabilization via repeated thermal cycling. Commercial paraffin wax (melting range: 52–54 °C; latent heat: 210 kJ/kg) was initially heated to 70 °C in a temperature-controlled water bath to ensure complete melting. Silver nanoparticles (Ag, 99.9% purity, average diameter: 40 nm, spherical morphology) were separately dispersed in ethanol at a concentration of 10 mg/mL to promote uniform distribution.

Paraffin wax was selected as the base phase change material (PCM) due to its favorable thermophysical properties, chemical stability, non-toxicity, and cost-effectiveness. Its melting temperature range (52–54 °C) closely matches the typical operating conditions of solar still systems, enabling efficient thermal energy storage during peak solar radiation periods and controlled heat release during off-peak hours.

Compared to other nano-enhanced phase change materials (NePCMs) reported in the literature^40^, including salt hydrates, fatty acids, and advanced composite systems, paraffin-based PCMs exhibit superior long-term stability, negligible phase separation, and minimal subcooling effects. In contrast, many alternative NePCM systems, although capable of providing higher thermal conductivity, may face challenges related to stability, material compatibility, and cost, which can limit their practical applicability in long-term solar thermal applications.

As reported in^40^, a wide range of advanced NePCMs have been investigated for solar still applications, demonstrating significant improvements in freshwater productivity. However, the review also indicates that performance enhancement alone is not sufficient, and practical deployment requires careful consideration of system stability, economic feasibility, and long-term operational reliability. In this context, the selection of paraffin as the base PCM represents a robust and practical choice.

To overcome the inherently low thermal conductivity of paraffin wax, silver nanoparticles were incorporated at a concentration of 1 wt% to enhance heat transfer characteristics and accelerate the thermal response during both charging and discharging processes. This concentration was selected based on previous studies indicating optimal enhancement in thermal conductivity without significant agglomeration. The nanoparticle suspension was added dropwise into the molten paraffin under continuous magnetic stirring at 300 rpm, and the mixture was maintained at 70 °C for 2 h to ensure homogeneous dispersion.

Subsequently, ethanol was removed by maintaining the mixture at 80 °C under atmospheric conditions with continuous stirring for an additional 3 h, ensuring complete evaporation while preserving nanoparticle uniformity. The resulting PCM–Ag composite was then subjected to five thermal cycles between 30 and 70 °C to enhance stability and minimize nanoparticle agglomeration. Finally, after cooling to room temperature, the solidified composite was encapsulated within an aluminum enclosure positioned behind the corrugated absorber.

Overall, the selection of paraffin–Ag in this study represents a balanced trade-off between thermal performance enhancement, system durability, and economic feasibility, rather than solely maximizing thermal conductivity. This makes it a practical and reliable choice for solar distillation applications, particularly in decentralized and resource-limited regions, as supported by previous literature^40^.

### Instrumentation and measurements

Solar radiation intensity was measured using a pyranometer (range 0–2000 W/m^2^, accuracy ± 2%) mounted horizontally adjacent to the still installations at the same elevation. Water temperatures inside the still basin were monitored using K-type thermocouples (accuracy ± 0.5 °C) positioned at mid-depth location to represent bulk water temperature. Glass cover temperatures were measured using additional K-type thermocouples attached to the interior glass surface at multiple locations, with readings averaged to account for spatial variation. Ambient air temperature was recorded using a shielded thermocouple positioned 1.5 m above ground level in a ventilated enclosure to avoid direct solar radiation effects.

Wind speed was monitored using a cup anemometer (range 0–30 m/s, accuracy ± 0.3 m/s) mounted at 2 m height adjacent to the experimental setup. All temperature and radiation sensors were connected to a multi-channel data acquisition system with 16-bit resolution, recording measurements at 5-min intervals throughout each test day. The data acquisition system was programmed to calculate and store instantaneous values along with hourly and daily averages.

Freshwater productivity was quantified by collecting condensate from the still’s drainage channel in graduated cylinders. Hourly measurements were performed by recording accumulated volume at the end of each hour, then emptying the collection cylinder to begin the next measurement interval. Daily productivity was calculated as the sum of hourly measurements over the operational period from 7:00 to 18:00. Each configuration was tested over three consecutive clear-sky days, with results averaged to account for day-to-day variations in environmental conditions.

### Experimental procedure and monitoring

All experimental tests were conducted at the same outdoor location of Kafrelsheikh (Latitude: 31° 6′ 22.75″ N & Longitude: 30° 56′ 31.11″ E), Egypt during the period from June to August 2025 to ensure comparable solar conditions. Testing was limited to days with clear or nearly clear sky conditions (cloud cover < 10%) to minimize variability from atmospheric attenuation. Each configuration was operated under identical procedures to ensure fair comparison.

Daily operation commenced at 7:00 AM when the still basins were filled with brackish water (salinity 5000 ppm) to standardized depths: 40 mm for CSS and MSPSS-VFA, and 5 mm for corrugated absorber configurations (sufficient to wet the jute cloth surface completely). The tracking system was activated in automatic mode for tracked configurations, while untracked systems remained in fixed orientation. Initial measurements of water temperature, glass temperature, ambient temperature, solar radiation, and wind speed were recorded at 7:00 AM to establish baseline conditions.

Monitoring continued at 30-min intervals throughout the day until 18:00 when solar radiation dropped below 50 W/m^2^. Freshwater collection measurements were performed hourly by recording accumulated volume and emptying collection cylinders. At the conclusion of each test day, final measurements were recorded, and the still was drained and cleaned in preparation for the next day’s testing. Each configuration underwent testing for 3 days under comparable conditions, with daily results averaged to determine representative performance values.

### Data analysis and performance metrics

System performance was characterized using multiple metrics to enable comprehensive evaluation. Daily productivity was calculated as the total volume of freshwater collected over the operational period, normalized by the still’s base area (0.6 m^2^) and expressed in mL/m^2^.day. Hourly productivity was similarly calculated for each 1-hour interval and expressed in mL/m^2^.hr.

Temperature differences between water and glass cover were calculated as indicators of evaporation potential, with larger differences generally indicating higher driving forces for evaporation and condensation processes. Peak temperatures and their timing were identified to assess thermal response characteristics.

Percentage improvements were calculated relative to CSS baseline performance to quantify enhancement effectiveness. Individual enhancement contributions were estimated by comparing sequential configurations: vertical absorber effect (MSPSS-VFA vs. CSS), single-axis tracking effect (MSPSS-STVFA vs. MSPSS-VFA), dual-axis tracking effect (MSPSS-DTFA vs. MSPSS-VFA), corrugated absorber effect (MSPSS-DTCA vs. MSPSS-DTFA), and PCM integration effect (MSPSS-DTCA + PCM-Ag vs. MSPSS-DTCA).

1 $$\text{Productivity\,Enhancement\,Ratio (PER)}: PER\:=\frac{{P}_{enhanced}\:-\:{P}_{baseline}}{{P}_{baseline}}\:\times\:100\%$$

where $P$ represents daily productivity for enhanced and baseline configurations.

The thermal efficiency of each solar still configuration was calculated to assess the effectiveness of converting incident solar energy into useful distillation output. The instantaneous thermal efficiency (η) was determined using the following relationship:

2 $$\eta\:\:=\frac{\dot{m}\:\times\:\:{h}_{fg}}{I\:\times\:\:A}\:\times\:100\%$$

where: $\dot{m}$ is the mass flow rate of distilled water production (kg/s). ${h}_{fg}$ is the latent heat of vaporization of water (2260 kJ/kg at atmospheric pressure). I is the incident solar radiation intensity (W/m^2^). A is the absorber surface area (m^2^).

For daily efficiency calculations, the cumulative energy required for distillation was compared to the total incident solar energy over the operational period:

3 $${\eta}_{daily}=\frac{{M}_{daily}\:\times\:\:{h}_{fg}}{\int\:I\left(t\right)dt\:\times\:\:A}\times\:100\%$$

where: ${M}_{daily}$ is the total daily freshwater production (kg/day). $\int\:I\left(t\right)dt$ represents the integrated solar radiation over the day (J/m^2^).

The overall thermal efficiency accounts for all energy conversion steps including solar radiation absorption, heat transfer to water, evaporation, vapor transport, and condensation. This metric provides insight into how effectively the system utilizes available solar energy and enables comparison between different configurations on an energy basis rather than purely productivity basis.

### Economic analysis

A comprehensive techno-economic analysis was performed to evaluate the financial viability of the enhanced solar still configurations. The analysis incorporated capital costs, operational expenses, system lifespan, and productivity to determine the levelized cost of freshwater production. The methodology follows established engineering economics principles commonly applied to renewable energy systems and desalination technologies.

Detailed capital cost analysis was performed for both conventional solar still (CSS) and the fully enhanced configuration (MSPSS-DTCA + PCM-Ag) to evaluate economic viability. Component costs were estimated based on current market prices for materials and standard labor rates for fabrication and assembly work. Table 2 presents the detailed cost breakdown.

**Table 2:** Component costs for CSS and MSPSS-DTCA + PCM-Ag.

Component	CSS cost ($)	MSPSS-DTCA + PCM-Ag cost ($)
Steel sheet	20	15
Glass cover	10	25
Fittings	20	20
Painting	5	5
Insulation	10	5
Fabrication fees	35	60
Tracking circuit	–	50
PCM	–	10
Silver nanoparticles	–	30
Total fixed cost (F)	100	220

A comprehensive levelized cost analysis was performed using standard engineering economic methods to determine the cost per liter of freshwater produced over the system lifetime. The analysis incorporated capital costs, maintenance expenses, salvage value, and productivity to calculate equivalent annual costs that account for time-value of money effects. Table 3 presents the economic analysis parameters and assumptions.

**Table 3:** Economic analysis parameters and assumptions.

Parameter	Symbol	Value	Unit
System lifespan	$$\:n$$	10	Years
Interest rate	$$\:i$$	15	%
Operating days per year	$$\:N$$	330	days/year
Fixed capital cost	$$\:F$$	100 (CSS) vs. 220 (MSPSS)	$

Key economic calculations were performed using standard formulas presented in Table 4.

**Table 4:** Economic analysis calculation methods^41–43^.

Parameter	Formula	Definition
Capital recovery factor	$\:CRF=\frac{i\:{(1+i)}^{n}}{{(1+i)}^{n}-1}$	Converts capital cost to equivalent annual cost
Fixed annual cost	$\:FAC=F\:\left(CRF\right)$	Annual cost equivalent of initial investment
Sinking fund factor	$\:SFF=\frac{i\:}{{(1+i)}^{n}-1}$	Converts future salvage value to annual equivalent
Salvage value	$\:S=0.2\:F$	Residual value at end of lifespan
Annual salvage value	$\:ASV=\mathrm{S}\:\left(\mathrm{S}\mathrm{F}\mathrm{F}\right)$	Annual equivalent of salvage value
Annual maintenance cost	$\:AMC=0.15\:\left(\mathrm{F}\mathrm{A}\mathrm{C}\right)$	Yearly maintenance and repairs
Total annual cost	$\:TAC=\mathrm{F}\mathrm{A}\mathrm{C}+\mathrm{A}\mathrm{M}\mathrm{C}-\mathrm{A}\mathrm{S}\mathrm{V}$	Net annual cost
Cost per liter	$\:CPL=TAC/M$	Levelized cost of freshwater

## Results and discussion

### Performance of CSS and SPSS with horizontal absorber

**Fig. 5:**
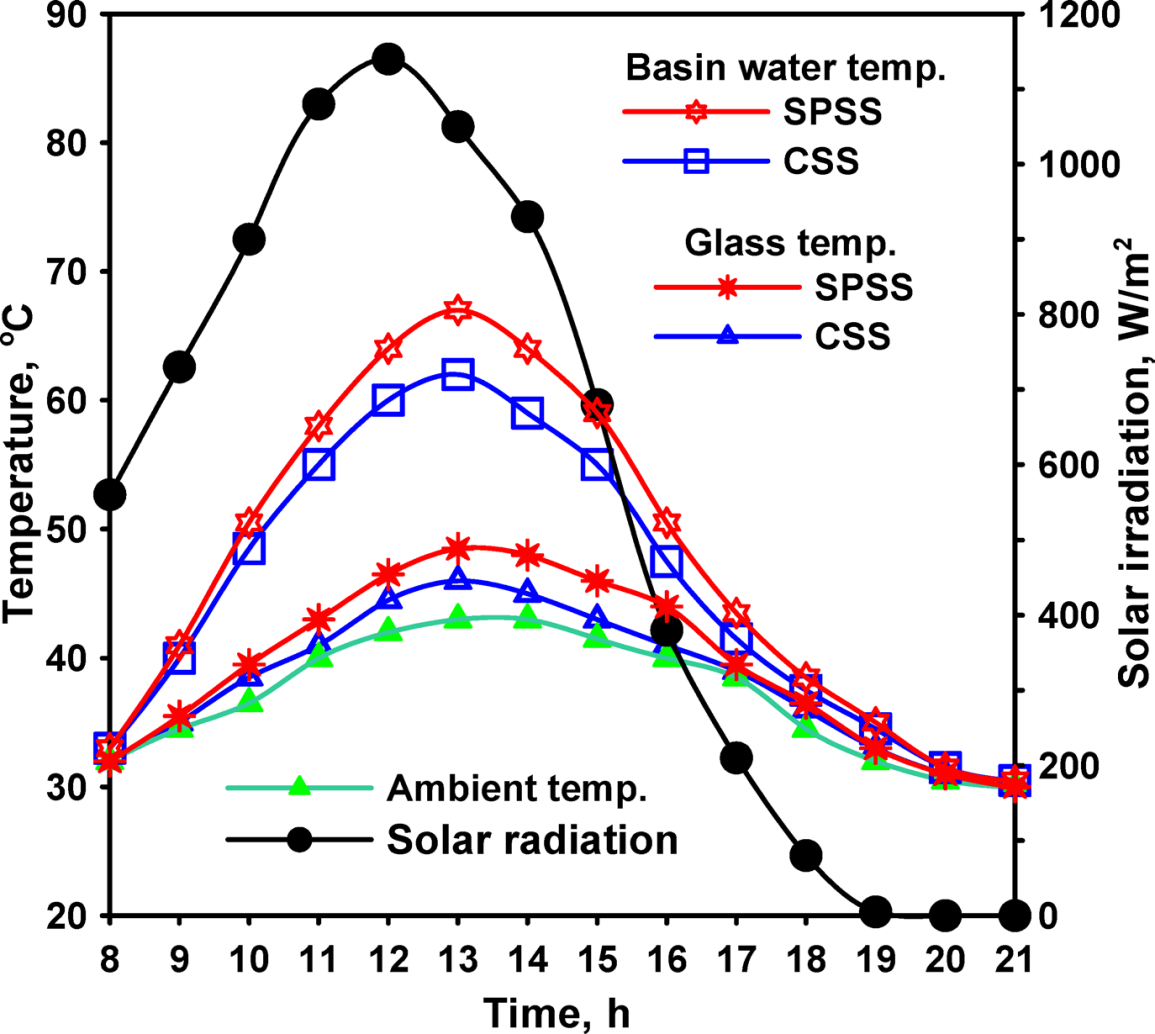
Solar radiation and temperatures for the distillers’ parts of glass and water.

Initial baseline testing compared conventional spherical solar still (CSS) performance with a basic spherical solar still with horizontal absorber (SPSS) without enhancements. Environmental conditions during this testing phase showed peak average solar radiation intensity of 1050 W/m^2^ occurring at 12:00, with ambient air temperatures ranging from 29 to 43 °C throughout the day as shown in Fig. 5. Wind velocity varied between 0.2 and 4.6 m/s, representing typical summer conditions at the test location.

Thermal performance data revealed that SPSS achieved higher water temperatures than CSS throughout the operational period. At 13:00 (near peak solar radiation), water temperatures reached 67 °C for SPSS compared to 62 °C for CSS, representing a 5 °C difference favoring the spherical configuration. This temperature advantage was attributed to the reduced shading effects in the spherical geometry compared to conventional basin-type stills. In CSS, the basin walls cast shadows on the water surface during morning and evening hours when solar elevation angles are low, reducing effective radiation absorption. The spherical SPSS configuration minimized these shading effects, allowing more uniform radiation distribution across the water surface throughout the day.

Glass cover temperatures exhibited similar trends but with smaller absolute differences between configurations. At 13:00, glass temperatures measured 49 °C for SPSS and 46 °C for CSS, a difference of 3 °C. The relatively smaller temperature differential at the glass surface was attributed to the substantially larger glass area in SPSS (2 m^2^) compared to CSS (0.6 m^2^), which provided greater heat dissipation capacity and maintained lower average temperatures despite higher evaporation rates.

**Fig. 6:**
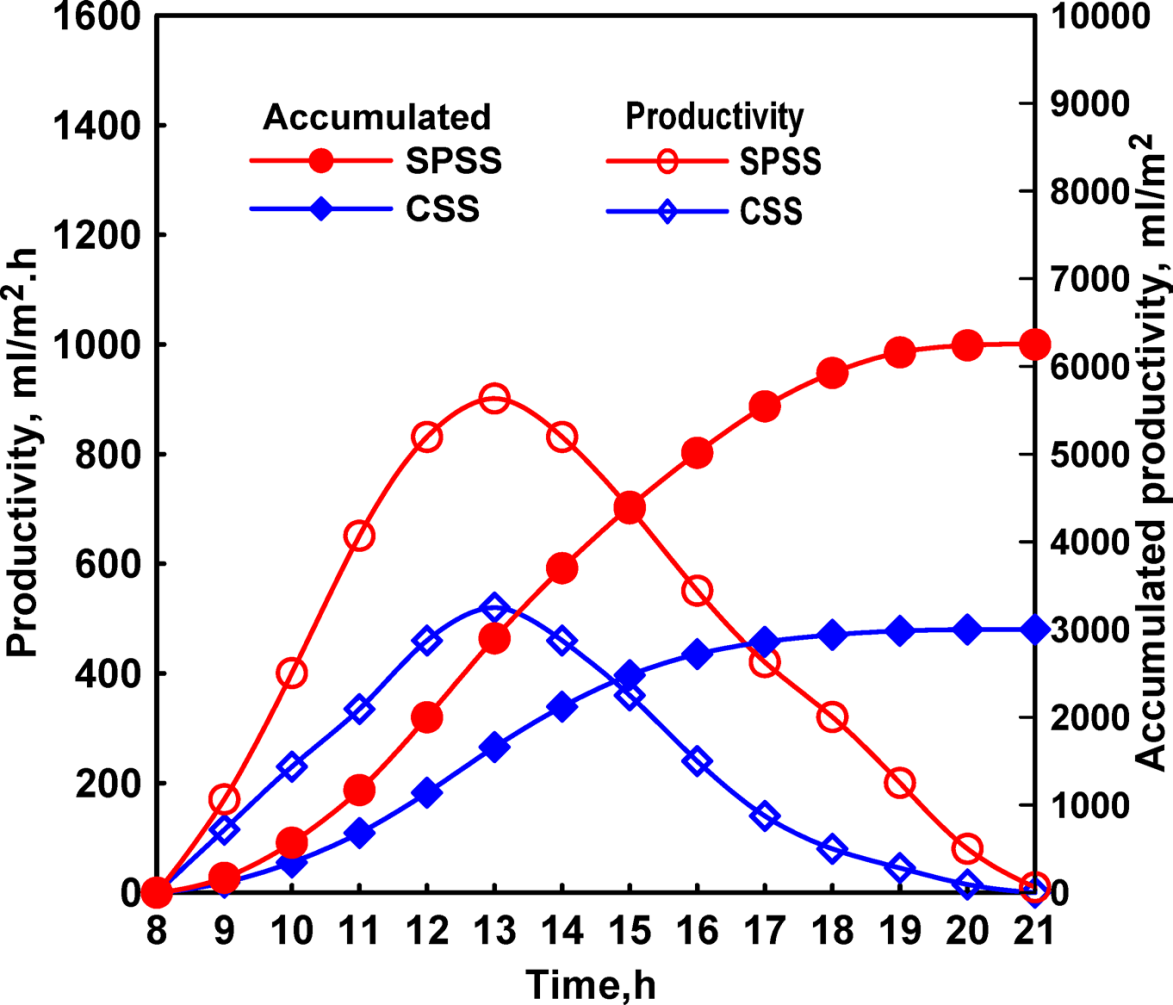
Both the hourly and total distillates of CSS and SPSS.

Productivity measurements showed clear advantages for the spherical configuration, Fig. 6. Hourly freshwater production peaked at 13:00 with values of 900 mL/m^2^.hr for SPSS compared to 520 mL/m^2^.hr for CSS. The cumulative daily production reached 6,250 mL/m^2^.day for SPSS and 3000 mL/m^2^.day for CSS, representing 108% higher productivity for the spherical design. This substantial improvement was attributed to three synergistic factors: reduced shading effects enabling more complete solar radiation utilization, larger condensation surface area facilitating more efficient vapor collection, and the favorable geometry that promotes natural convective circulation enhancing both evaporation and condensation processes.

The larger glass area in SPSS provided extended condensation surface area, which is critical for maintaining high productivity rates. As water vapor rises from the heated basin, it encounters the cooler glass surface where condensation occurs. Insufficient condensation area can create bottlenecks limiting overall productivity, as saturated vapor prevents further evaporation from the water surface below. The 233% increase in glass area (from 0.6 m^2^ to 2 m^2^) provided ample condensation capacity, ensuring that evaporation rates rather than condensation capacity became the limiting factor in freshwater production.

### Performance of CSS and MSPSS with fixed vertical flat absorber (MSPSS-VFA)

**Fig. 7:**
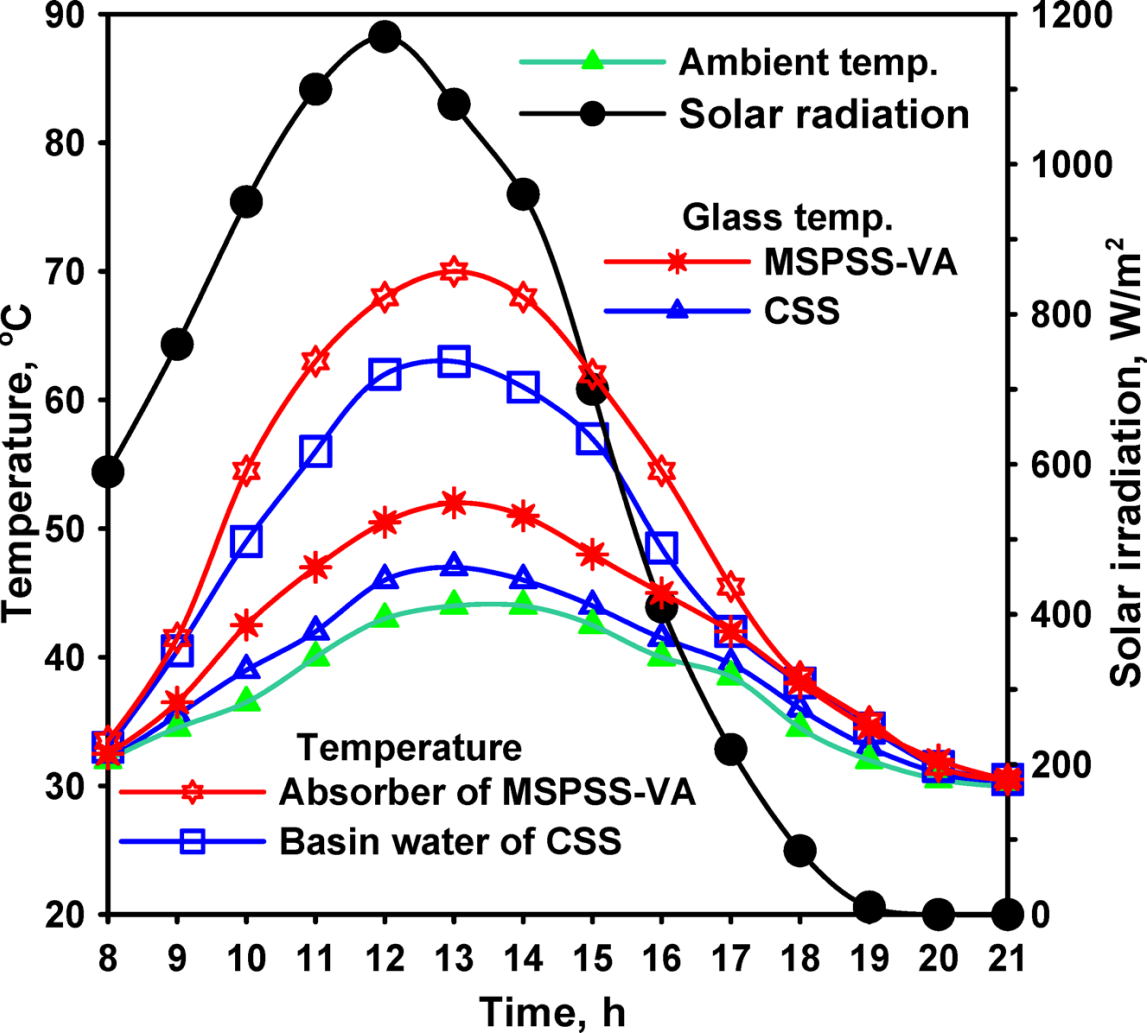
Solar radiation and temperatures for the distillers’ parts of glass and water for both CSS and MSPSS-VA.

The introduction of a vertical flat absorber wrapped with jute cloth represented a significant modification aimed at reducing water inventory while increasing effective evaporation surface area. Environmental conditions, shown in Fig. 7, during this testing phase showed peak average solar radiation of 1170 W/m^2^ at 12:00, with ambient temperatures ranging from 39 to 44 °C, representing slightly more intense conditions than the previous test series.

Thermal measurements at 13:00 showed glass cover temperatures of 47 °C for CSS and 52 °C for MSPSS-VFA, representing a 5 °C increase for the modified configuration. Water temperatures measured 63 °C for CSS and 70 °C for MSPSS-VFA, a difference of 7 °C favoring the vertical absorber design. These elevated temperatures resulted from several factors associated with the vertical absorber configuration. The thin water film maintained on the jute cloth surface through capillary action minimized thermal mass, enabling more rapid temperature response to solar radiation input. The reduced water inventory (approximately 0.5 L compared to 4 L for horizontal configurations) meant that less energy was required to achieve a given temperature rise, accelerating the heating process during morning hours.

The vertical orientation also improved radiation absorption geometry during morning and evening hours when the sun’s elevation angle is low. Horizontal absorbers receive radiation at grazing incidence angles during these periods, increasing reflection losses and reducing effective absorption. The vertical absorber maintained more favorable incident angles throughout the day as it could be positioned to better intercept radiation at different times.

**Fig. 8:**
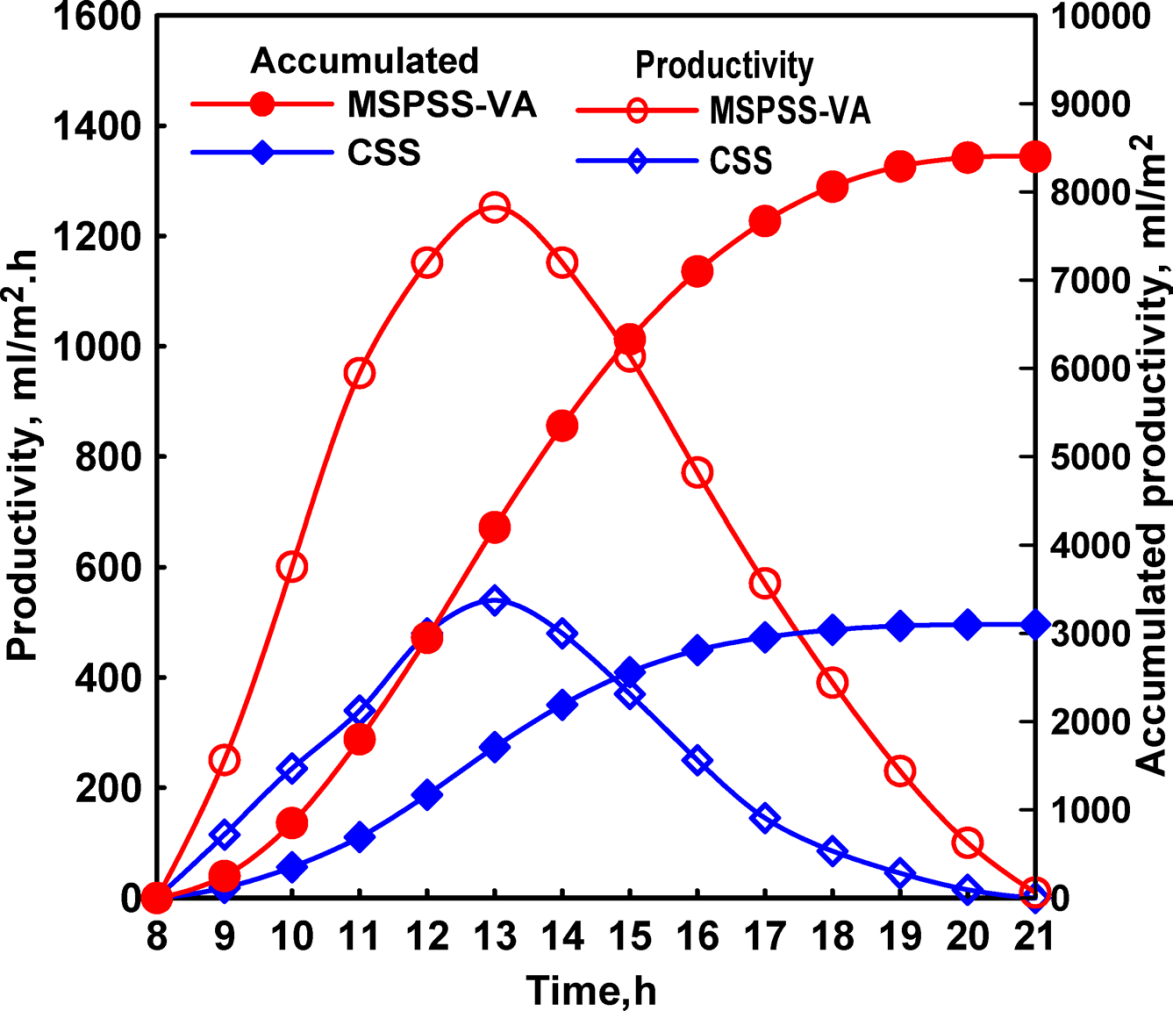
Both the hourly and total distillates of CSS and MSPSS-VA.

Productivity results demonstrated substantial benefits from the vertical absorber modification. Hourly production peaked at 1,250 mL/m^2^.hr at 13:00 for MSPSS-VFA compared to 540 mL/m^2^.hr for CSS as obtained in Fig. 8. Daily cumulative production reached 8,400 mL/m^2^.day for MSPSS-VFA compared to 3,100 mL/m^2^.day for CSS, representing 171% improvement. Comparing this result to the previous SPSS configuration (108% improvement), the incremental benefit of changing absorber orientation from horizontal to vertical was approximately 62%, highlighting the significant impact of this single modification.

The jute cloth wicking material played a crucial role in maintaining uniform water distribution across the vertical absorber surface. Without the capillary wicking action, water would drain rapidly under gravity, leaving portions of the absorber surface dry and reducing effective evaporation area. The jute cloth absorbed water and distributed it uniformly through capillary forces, maintaining a thin wetted film across the entire absorber height. This maximized evaporation surface area while minimizing water depth, combining the benefits of increased surface area with reduced thermal mass.

### Performance of MSPSS-STVFA with single-axis tracking vertical flat absorber

Implementation of single-axis solar tracking provided additional performance enhancement by maintaining more favorable absorber orientation to incoming solar radiation throughout the day. The single-axis tracker adjusted the absorber’s east-west (azimuthal) position to follow the sun’s daily trajectory from sunrise to sunset, but did not adjust elevation angle, which remained fixed at an optimized position.

Environmental conditions during this testing phase showed peak solar radiation of 1250 W/m^2^ at 12:00, with ambient temperatures ranging from 44.5 to 45 °C. Thermal measurements at 13:00 revealed water temperatures of 62 °C for CSS and 71 °C for MSPSS-STVFA, representing a 9 °C differential. Glass cover temperatures measured 48 °C for CSS and 54 °C for MSPSS-STVFA, a 6 °C difference.

The enhanced temperatures achieved with tracking were attributed to the improved radiation interception throughout the day. By maintaining near-perpendicular orientation to incident sunlight, the tracked absorber minimized reflection losses and maximized energy absorption. This effect was particularly pronounced during morning and evening hours when the sun’s position differs substantially from the fixed absorber’s optimal midday orientation. During these off-peak hours, tracking provided the greatest incremental benefit, effectively extending the productive operating period.

Productivity measurements showed daily cumulative production of 10,300 mL/m^2^.day for MSPSS-STVFA compared to 3250 mL/m^2^.day for CSS, representing 217% improvement. By comparing with the fixed vertical flat absorber configuration (171% improvement), the incremental benefit attributable specifically to single-axis tracking was approximately 46%. This substantial improvement demonstrated the value of active tracking systems for solar distillation applications, confirming findings from photovoltaic research that tracking significantly enhances energy collection.

The temporal distribution of productivity gains provided additional insights into tracking system benefits. During midday hours (10:00–14:00) when the sun’s position approximates the fixed absorber’s optimal orientation, productivity differences between tracked and untracked systems were relatively modest (approximately 15–20% advantage for tracking). However, during morning hours (7:00–10:00) and afternoon hours (14:00–18:00), the tracked system maintained 40–60% higher productivity than the untracked configuration. This temporal pattern confirmed that tracking primarily benefited performance during off-peak hours by maintaining favorable radiation interception geometry.

### Performance of MSPSS-DTFA with dual-axis tracking flat absorber

Advancing from single-axis to dual-axis tracking added elevational adjustment capability, enabling the absorber to maintain perpendicular orientation to solar radiation in both azimuthal and elevational dimensions. This comprehensive tracking provided optimal alignment throughout the day and across seasons, maximizing radiation interception under all conditions.

**Fig. 9:**
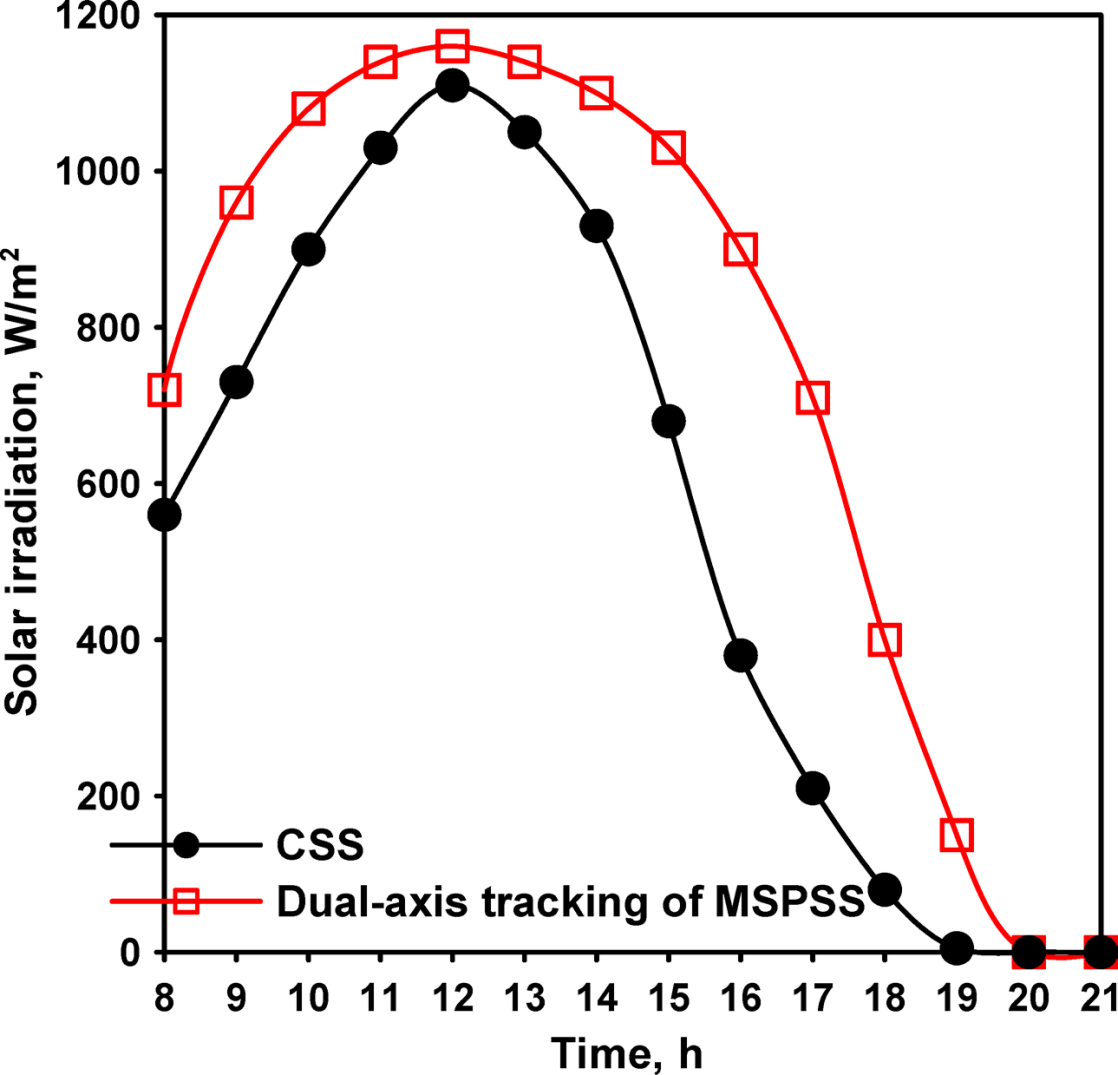
The solar radiation incident on both CSS and MSPSS with dual-axis solar tracking.

Comparative measurements of solar radiation intensity demonstrated the benefits of dual-axis tracking. The solar radiation incident on both CSS and MSPSS with dual-axis solar tracking is illustrated in Fig. 9. Throughout the day, the radiation intensity measured by a sensor mounted on the tracked absorber consistently exceeded the intensity measured by a horizontal reference sensor, confirming that the tracking system successfully maintained favorable orientation. The period of maximum intensity extended from 10:00 to 17:00 for the tracked system, compared to a narrower peak period (11:00–15:00) for fixed systems, effectively broadening the productive operating window.

**Fig. 10:**
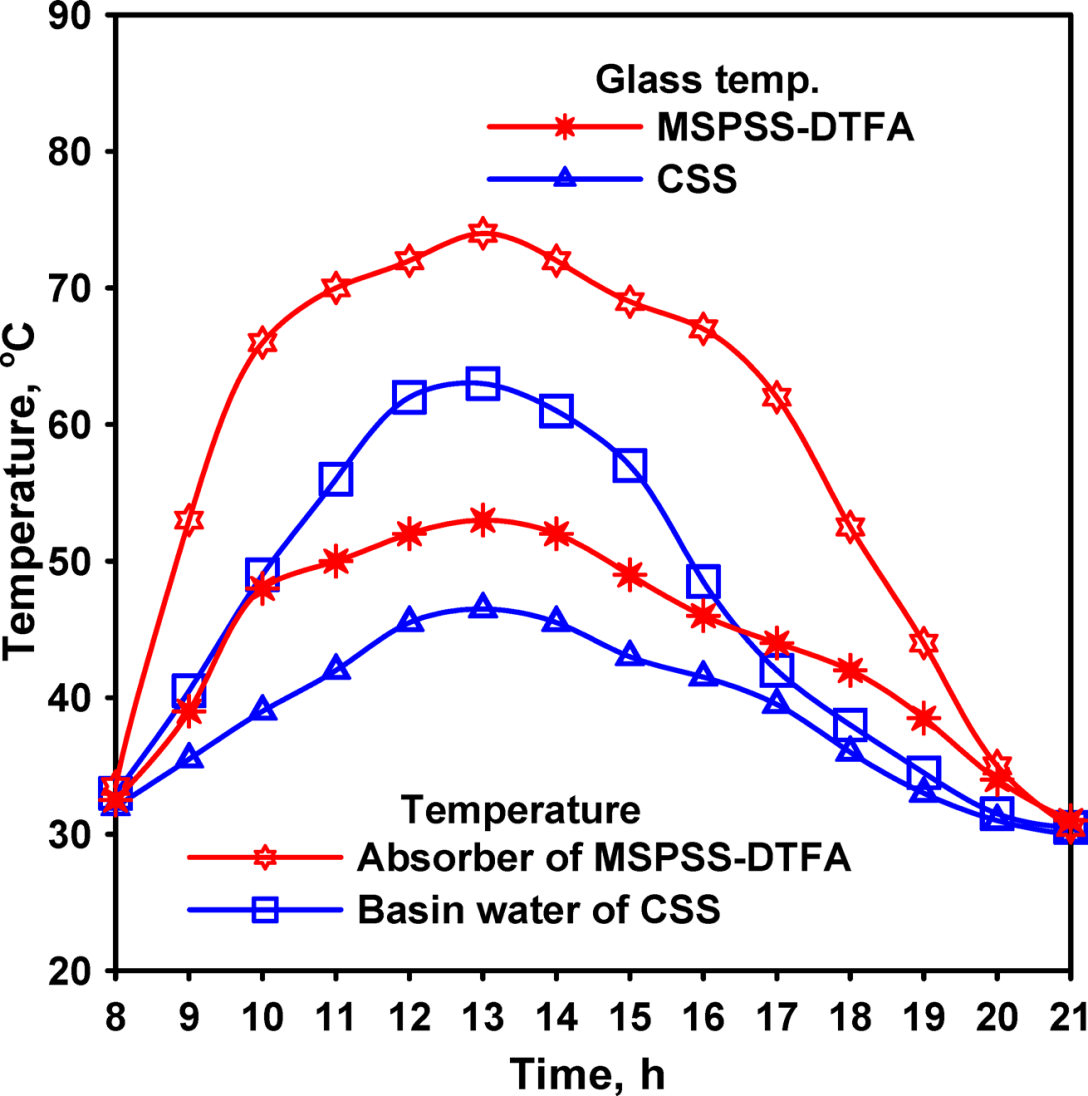
The glazing and water temperatures for CSS and MSPSS with dual-axis solar tracking.

Thermal data showed that both water and glass temperatures for the tracked system followed the temporal pattern of incident radiation on the tracked surface rather than the pattern for horizontal surfaces. Water temperatures reached 63 °C for CSS and 74 °C for MSPSS-DTFA at peak conditions, representing an 11 °C differential as presented in Fig. 10. Glass temperatures measured 46.5 °C for CSS and 53.5 °C for MSPSS-DTFA, a 7 °C difference. These sustained elevated temperatures throughout the extended high-radiation period contributed to enhanced productivity.

**Fig. 11:**
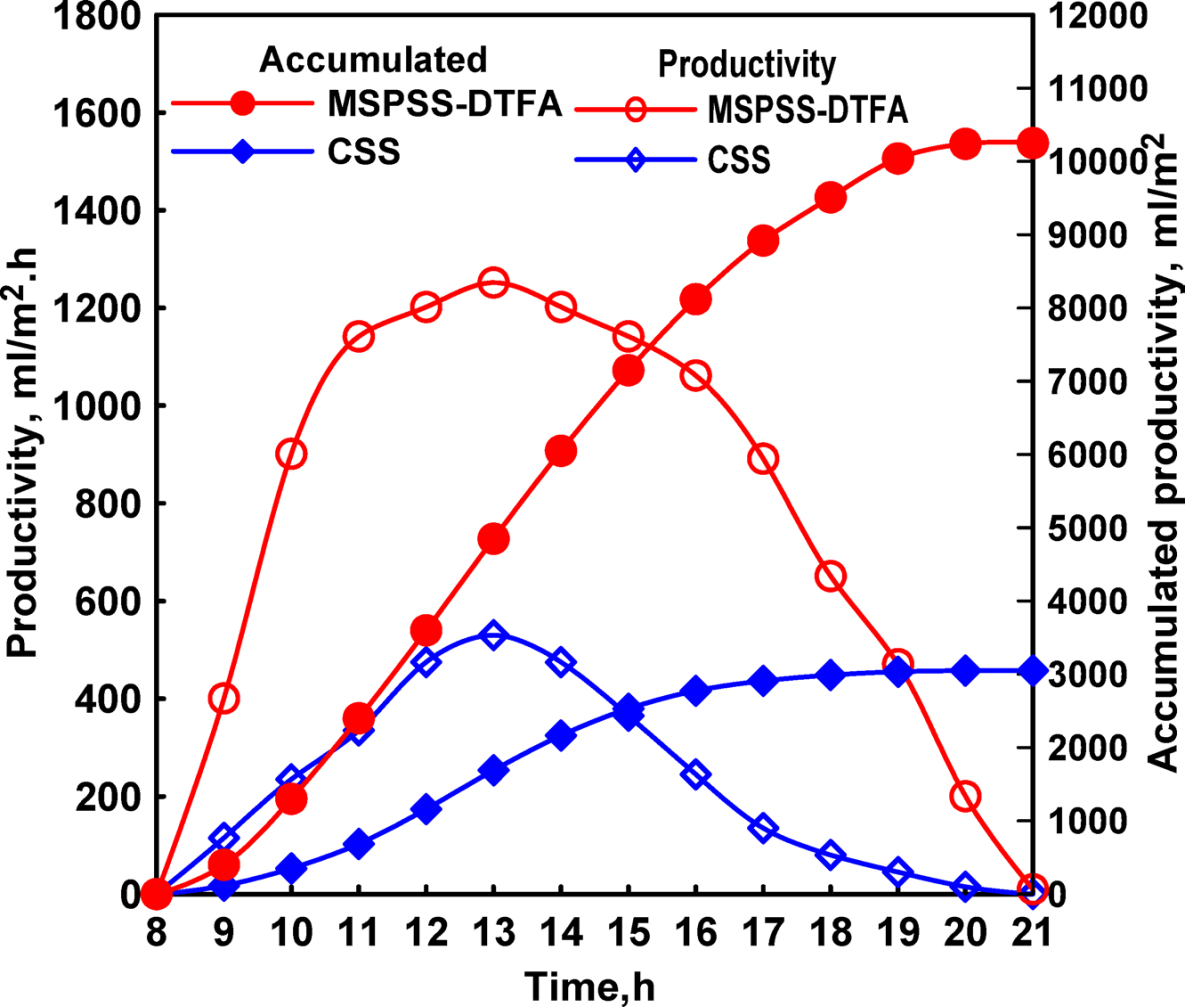
Both the hourly and total distillates of CSS and MSPSS with dual-axis solar tracking.

Figure 11 presents the hourly and total distillates of CSS and MSPSS with dual-axis solar tracking. Daily productivity measurements revealed cumulative production of 10,250 mL/m^2^.day for MSPSS-DTFA compared to 3,050 mL/m^2^.day for CSS, representing 236% improvement over the baseline configuration. Comparing with the fixed vertical flat absorber baseline (171% improvement), the incremental benefit specifically from dual-axis tracking was approximately 65%. This represented a substantial improvement over single-axis tracking (46% benefit), demonstrating the value of complete two-dimensional tracking capability.

The additional benefit of dual-axis versus single-axis tracking (approximately 19% points) was attributed primarily to elevational angle optimization during morning and evening hours. During these periods, the sun’s elevation angle differs significantly from midday values, and single-axis systems cannot compensate for this variation. The dual-axis system adjusted both azimuth and elevation continuously, maintaining near-optimal orientation throughout the entire operational period.

The enhanced performance during shoulder hours (early morning and late afternoon) proved particularly valuable for overall daily productivity. While these periods contribute relatively less energy than midday hours in absolute terms, their cumulative contribution over the full operational period (7:00–18:00) is substantial. By maintaining high efficiency during these extended hours, the dual-axis tracking system effectively increased the duration of productive operation, compensating somewhat for lower absolute radiation intensities during these times.

### Performance of MSPSS-DTCA with dual-axis tracking corrugated absorber

The introduction of corrugated absorber geometry represented another major enhancement aimed at increasing effective evaporation surface area. The sinusoidal corrugation pattern increased surface area by approximately 86% compared to the equivalent flat absorber while maintaining the same projected frontal area and overall dimensions. This geometric modification provided additional evaporation area without requiring larger still footprints or increased material costs.

The corrugated surface, when wrapped with jute cloth, maintained thin water films across the entire expanded surface area through capillary wicking action. The increased perimeter and surface complexity enhanced capillary distribution, ensuring uniform wetting across peaks and valleys of the corrugation pattern. This complete wetting maximized the benefit of the increased geometric surface area.

Thermal characteristics of the corrugated absorber configuration showed elevated temperatures consistent with the increased evaporation rate. Water temperatures reached 74 °C for MSPSS-DTCA compared to 63 °C for CSS at peak conditions. Glass cover temperatures measured 53.5 °C for the corrugated configuration, representing elevated values that reflected the intensified evaporation and condensation processes occurring within the still.

Daily productivity measurements demonstrated substantial benefits from the corrugated absorber modification. The MSPSS-DTCA configuration achieved 12,320 mL/m^2^.day compared to 3150 mL/m^2^.day for CSS, representing 291% improvement. Comparing with the flat absorber dual-tracking configuration (236% improvement), the incremental benefit specifically attributable to the corrugated geometry was approximately 55%. This significant improvement confirmed the value of geometric surface area enhancement for solar distillation applications.

The combined effect of all modifications to this point (vertical orientation, dual-axis tracking, and corrugated geometry) yielded 120% improvement compared to the vertical flat absorber without tracking (171% vs. 291%). This substantial enhancement demonstrated that multiple modifications can be combined effectively to achieve cumulative performance improvements far exceeding what any single enhancement could provide independently.

The corrugated geometry also promoted turbulent mixing within the thin water film, enhancing convective heat transfer compared to smooth flat surfaces where laminar boundary layers restrict heat transfer. This secondary benefit complemented the primary surface area enhancement, contributing to the observed productivity improvements. The enhanced mixing also helped maintain more uniform temperature distribution across the absorber surface, preventing localized hot or cold spots that could reduce overall efficiency.

### Performance of MSPSS-DTCA + PCM-Ag with phase change material integration

Despite the substantial improvements achieved through absorber modification and tracking, thermal analysis revealed a potential limiting factor: elevated glass cover temperatures resulting from intensified evaporation rates. Higher glass temperatures reduce the temperature differential between glass and ambient air, decreasing heat loss and slowing condensation rates. This thermal bottleneck could potentially limit the effectiveness of additional evaporation enhancement measures.

**Fig. 12:**
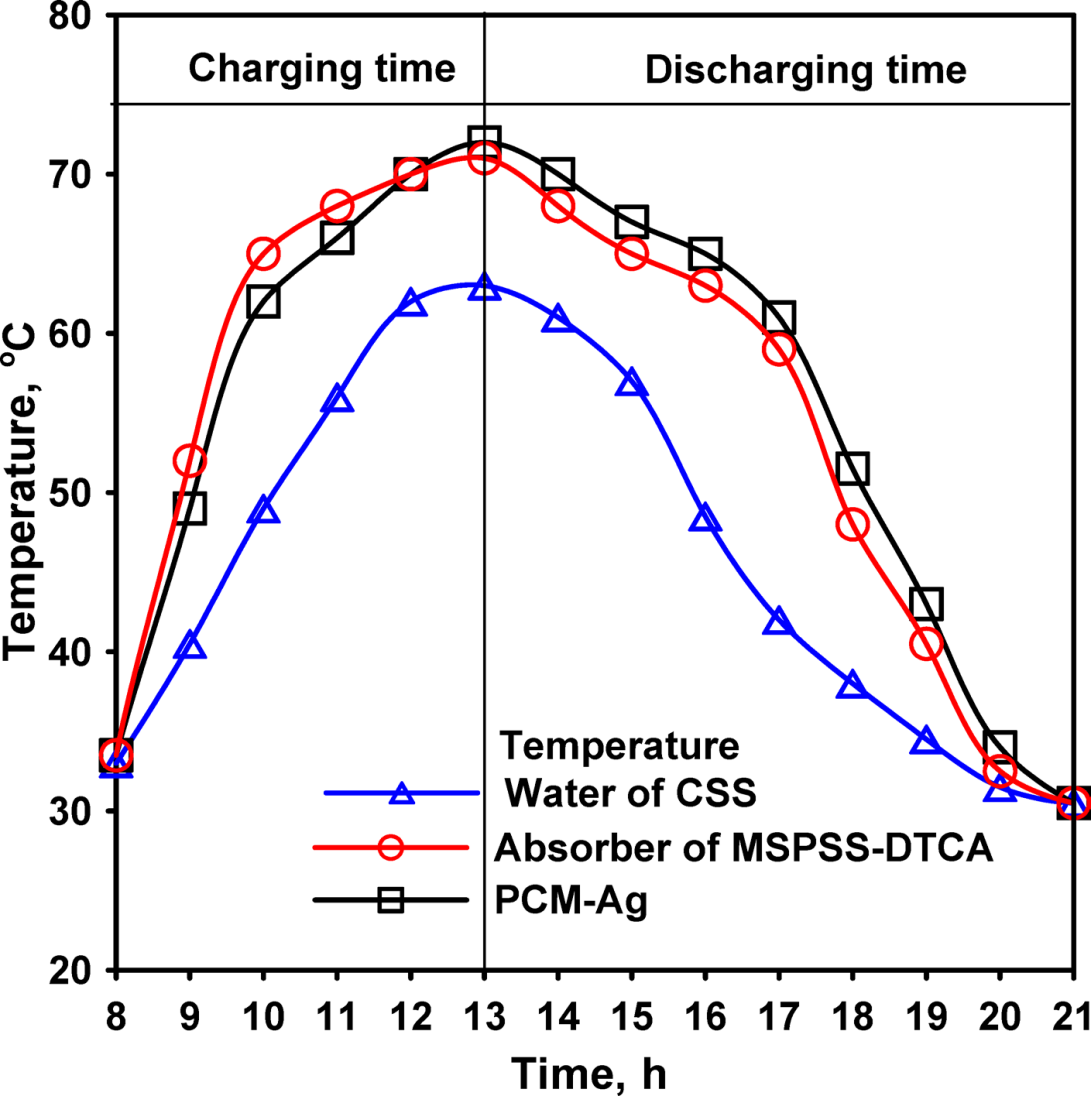
The water and absorber temperatures for CSS and MSPSS with dual-axis solar tracking and PCM.

Integration of silver nanoparticle-enhanced phase change material (PCM-Ag) addressed this thermal management challenge. The PCM was positioned in a 20 mm thick layer behind the corrugated absorber, providing thermal mass and temperature moderation. During periods of intense solar radiation and high temperatures (typically 10:00–15:00), the PCM absorbed excess thermal energy as latent heat during melting, limiting temperature rise at the absorber surface as shown in Fig. 12. This absorption helped moderate glass cover temperatures, maintaining favorable temperature gradients for condensation.

During periods of declining solar radiation (15:00–18:00 and into evening hours), the PCM released stored energy as it solidified, helping maintain elevated temperatures and extending productive operation beyond peak solar hours. This thermal storage effect smoothed temperature fluctuations and shifted some energy availability from peak to off-peak periods, potentially improving overall daily productivity.

The silver nanoparticle enhancement improved PCM thermal conductivity, accelerating both heat absorption (charging) and heat release (discharging) processes. Enhanced thermal conductivity ensured that the PCM could respond rapidly to temperature changes, maximizing its effectiveness for real-time thermal management rather than merely providing passive thermal mass. Literature reports indicate that 1% silver nanoparticle loading can increase paraffin thermal conductivity by 25–40%, substantially improving practical performance.

Thermal measurements confirmed the moderating effect of PCM integration. Glass cover temperatures for MSPSS-DTCA + PCM-Ag remained slightly lower than for MSPSS-DTCA alone during peak radiation hours, despite similar or higher productivity rates. This temperature moderation indicated improved thermal management, with the PCM effectively buffering extreme temperatures while maintaining favorable conditions for evaporation and condensation.

Daily productivity measurements showed exceptional performance for the fully enhanced configuration. MSPSS-DTCA + PCM-Ag achieved 13,300 mL/m^2^.day compared to 3,000 mL/m^2^.day for CSS, representing 343% improvement over the baseline conventional design. Comparing with the corrugated absorber tracking configuration without PCM (291% improvement), the incremental benefit specifically from PCM-Ag integration was approximately 52%. This represented a substantial contribution from thermal management enhancement, confirming that addressing thermal bottlenecks can yield significant productivity improvements.

The cumulative effect of all modifications (vertical orientation, dual-axis tracking, corrugated geometry, and PCM-Ag thermal management) transformed the solar still’s performance characteristics. The final configuration achieved 4.4 times the productivity of the conventional baseline design, demonstrating that comprehensive, integrated enhancement strategies can achieve dramatic performance improvements far exceeding incremental modifications.

System efficiency, defined as the ratio of energy required for freshwater production to incident solar energy, also improved substantially. While detailed efficiency calculations require precise energy balance measurements, the productivity improvements directly indicate enhanced efficiency in converting solar radiation to useful distillation output. The progressive modifications addressed multiple limiting factors systematically, eliminating bottlenecks and enabling more complete utilization of available solar energy.

### Comparative analysis of enhancement contributions

#### Productivity enhancement ratio (PER) of solar stills under various operating conditions

**Fig. 13:**
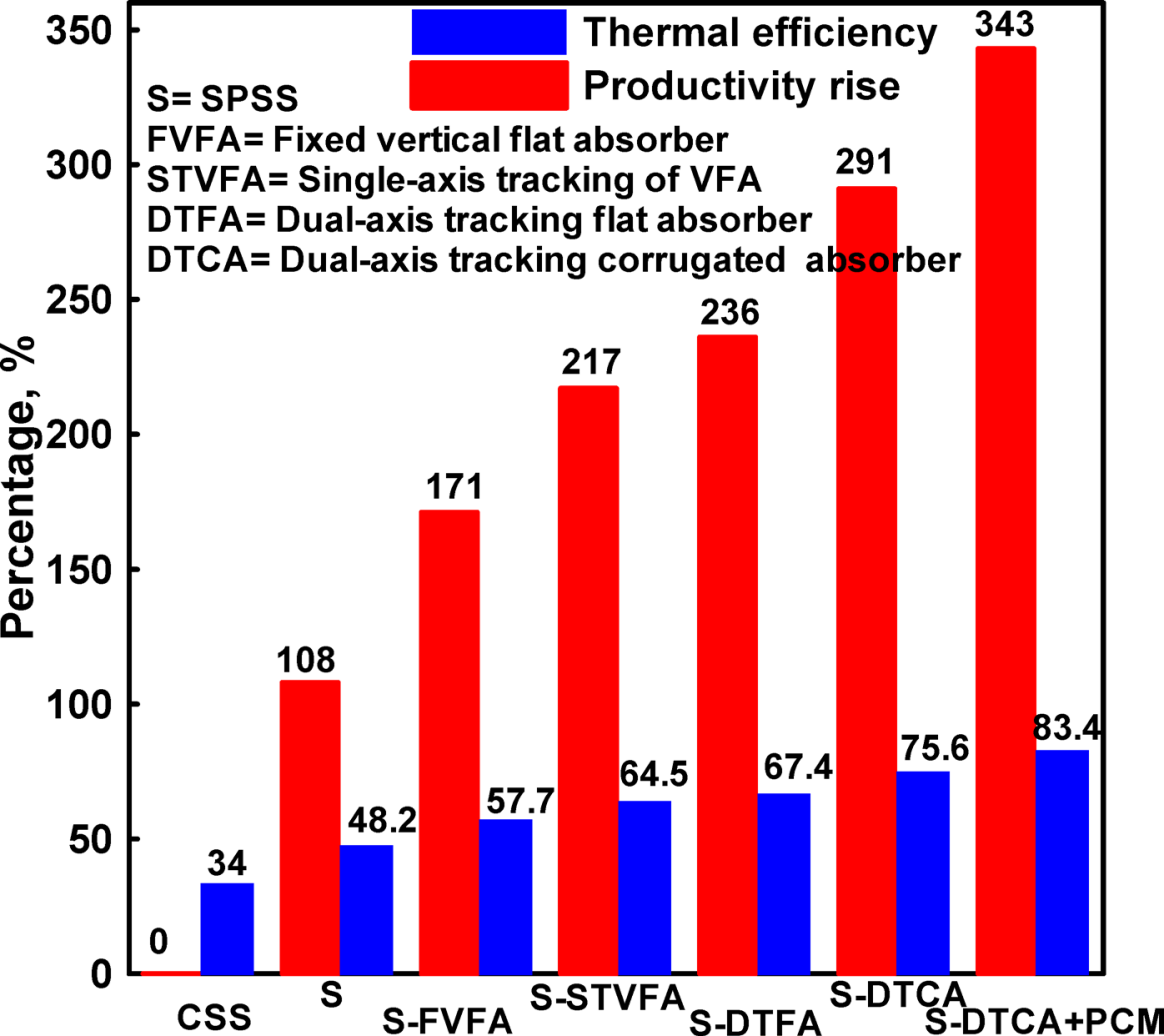
Productivity improvements and thermal efficiency attributable to each major modification relative to appropriate baseline configurations.

Systematic comparison of different configurations enables quantification of individual enhancement contributions and identification of synergistic interactions. Figure 13 summarizes the productivity improvements and thermal efficiency attributable to each major modification relative to appropriate baseline configurations. Several important observations emerge from this analysis. First, each individual enhancement provided substantial benefits, with improvements ranging from 46 to 65% relative to appropriate baselines. This demonstrates that multiple pathways exist for solar still performance improvement, and comprehensive enhancement requires addressing multiple factors rather than focusing on single modifications.

Second, the benefits of different enhancements appear largely additive rather than exhibiting strong negative interactions. The cumulative improvement from all modifications (343%) approximately equals the sum of individual contributions, suggesting that these enhancements address largely independent limiting factors. This additive behavior is encouraging for practical design optimization, as it indicates that combining multiple enhancement strategies will generally yield cumulative benefits.

Third, both passive modifications (corrugated geometry, PCM thermal management) and active systems (solar tracking) contributed significantly to overall performance improvement. This demonstrates that optimal solar still design requires both careful geometric and material optimization as well as active operational control. Passive enhancements alone cannot achieve maximum performance, nor can active tracking compensate fully for suboptimal geometric or thermal designs.

Fourth, thermal management through PCM integration provided benefits comparable to geometric enhancements (corrugated absorber), despite addressing a different limiting factor. This highlights the importance of considering thermal bottlenecks in addition to evaporation surface area when optimizing solar still designs. As evaporation rates increase through geometric or operational modifications, condensation and thermal management become increasingly important for realizing productivity improvements.

The dual-axis tracking improvement (65%) exceeded single-axis tracking (46%) by approximately 19% points, representing a 41% greater benefit for the more sophisticated tracking system. This substantial difference justifies the additional mechanical complexity and control requirements of dual-axis systems, particularly for applications where maximum productivity is prioritized over system simplicity. For cost-sensitive applications, single-axis tracking might represent a more appropriate compromise between performance and complexity.

#### Mechanistic role of solar tracking on solar energy utilization

The improvement in performance due to solar tracking can be attributed to enhanced utilization of incident solar radiation throughout the day. In fixed systems, the effective irradiance absorbed by the surface varies significantly with the sun’s position, leading to increased reflection losses during periods of high incidence angle, particularly in the morning and afternoon.

In contrast, the tracking system continuously adjusts the absorber orientation to maintain a near-optimal incidence angle, thereby maximizing the effective irradiance received by the surface. This results in a higher and more stable average absorbed solar energy over the entire operating period compared to fixed configurations.

The improvement is not solely due to higher instantaneous solar radiation, but rather the cumulative increase in absorbed energy over time. By reducing cosine losses and reflection losses, the tracking system extends the duration of high-efficiency operation and enhances overall thermal utilization.

Furthermore, dual-axis tracking provides additional improvement by correcting both azimuthal and elevational misalignment, ensuring near-perpendicular incidence conditions across different times of the day and seasonal variations. This further reduces cosine losses, particularly during early morning and late afternoon periods, where fixed systems experience pronounced performance degradation. However, the incremental benefit of dual-axis tracking is smaller compared to single-axis tracking, indicating diminishing returns with increasing system complexity.

#### Thermal efficiency of solar stills under various operating conditions

The thermal efficiency represents a critical performance metric that quantifies how effectively each solar still configuration converts incident solar radiation into useful distillation output. The thermal efficiency (η) was calculated using the relationship between the energy required for evaporation and the total solar energy incident on the absorber surface, as described in “Data analysis and performance metrics” section. This analysis provides insights into the fundamental energy conversion effectiveness of different enhancement strategies beyond simple productivity measurements (Fig. 13).

##### Baseline and horizontal absorber configurations

The conventional solar still (CSS) achieved a thermal efficiency of approximately 34%. This relatively modest efficiency is characteristic of passive basin-type solar stills and reflects several inherent limitations. Significant energy losses occur through: (1) bottom and side conduction losses through insulation, (2) convective and radiative losses from the glass cover to ambient environment, (3) thermal mass effects from the water inventory that stores energy without immediate productive use, and (4) reflection losses from non-optimal incident angles during morning and evening hours.

The spherical solar still with horizontal absorber (SPSS) demonstrated improved thermal efficiency of 48.2%, representing a 41.8% relative improvement over CSS. This substantial enhancement resulted from the spherical geometry’s advantages including reduced shading effects throughout the day, larger condensation surface area facilitating more efficient vapor collection, and improved radiation interception angles that reduced reflection losses. The curved glass cover maintained more favorable orientation to incident sunlight across a broader time period compared to the fixed-angle glass of CSS, enabling more complete utilization of available solar energy.

The 14.2% point absolute efficiency increase from CSS (34%) to SPSS (48.2%) corresponds well with the 108% productivity improvement observed between these configurations. This correlation confirms that the spherical geometry fundamentally improves energy conversion effectiveness rather than merely extending operating hours or benefiting from favorable weather conditions during testing.

##### Vertical absorber enhancement

The modified spherical solar still with fixed vertical flat absorber (MSPSS-VFA) achieved thermal efficiency of 57.7%, representing a 9.5% point improvement over SPSS and a 69.7% relative improvement over CSS. This efficiency gain resulted from multiple synergistic factors associated with the vertical absorber configuration.

The thin water film maintained on the vertical jute cloth surface through capillary action dramatically reduced the thermal mass compared to horizontal basin configurations. With water inventory reduced from approximately 4 L to 0.5 L, less solar energy was diverted into sensible heating of bulk water, allowing more energy to drive evaporation directly. This reduced thermal inertia enabled faster temperature response, allowing the system to reach productive operating temperatures earlier in the day and respond more quickly to variations in solar radiation intensity.

The vertical orientation also improved the effective utilization of solar radiation during off-peak hours. During morning and evening periods when the sun’s elevation angle is low, horizontal absorbers receive radiation at grazing incidence angles with high reflection losses. The vertical absorber could be oriented to receive radiation at more favorable angles during these periods, capturing energy that would otherwise be lost to reflection. This extended the period of high-efficiency operation beyond the narrow midday window typical of horizontal configurations.

Additionally, the jute cloth wicking material maximized the wetted surface area available for evaporation. Unlike horizontal water surfaces where evaporation occurs only at the air-water interface, the vertical cloth presented the entire absorber surface area for evaporation. This geometric enhancement increased the evaporation rate per unit of absorbed solar energy, directly improving thermal efficiency.

The 57.7% efficiency of MSPSS-VFA represents a significant achievement, approaching the theoretical limits for single-stage solar distillation without heat recovery or multi-effect configurations. This demonstrates that optimizing absorber geometry and water management can yield substantial efficiency improvements through passive design modifications alone.

##### Single-axis tracking enhancement

Implementation of single-axis tracking (MSPSS-STVFA) increased thermal efficiency to 64.5%, representing a 6.8% point improvement over the fixed vertical flat absorber configuration. This enhancement is attributed to improved solar energy capture through continuous adjustment of the absorber orientation along the east–west axis.

The observed improvement is most evident during morning and afternoon periods (7:00–10:00 and 15:00–18:00), where the system maintains higher energy absorption compared to the fixed configuration. During these shoulder hours, the tracked absorber achieved approximately 20–30% higher solar energy utilization, which directly contributed to increased evaporation rates and overall efficiency improvement.

During midday hours (10:00–14:00), the performance difference between tracked and fixed systems was relatively small (2–3% points), as both configurations operate near optimal alignment with incident solar radiation.

The performance enhancement can be further explained by improved effective solar irradiance on the absorber surface. In fixed configurations, cosine losses become significant at high incidence angles, particularly during morning and afternoon periods. The tracking system mitigates these losses by maintaining a more favorable orientation relative to the sun’s position, thereby increasing the time-averaged absorbed solar energy over the entire day. Consequently, the observed improvement is primarily driven by enhanced cumulative radiation utilization rather than instantaneous peak irradiance.

The achieved thermal efficiency of 64.5% demonstrates strong performance for a single-stage solar still with active tracking. This result highlights the effectiveness of integrating vertical absorber geometry, capillary wicking, and single-axis tracking to enhance overall solar energy utilization.

##### Dual-axis tracking enhancement

Advancing to dual-axis tracking (MSPSS-DTFA) further improved thermal efficiency to 67.4%, a 2.9% point gain over single-axis tracking. While this absolute improvement appears modest compared to previous enhancements, it represents valuable performance optimization for applications where maximum efficiency is prioritized.

Dual-axis tracking maintained near-perpendicular absorber orientation to incident sunlight in both azimuthal and elevational dimensions throughout the day. This comprehensive tracking capability minimized reflection losses under all conditions, enabling the absorber to capture the maximum possible fraction of incident solar radiation. The elevational tracking component proved particularly valuable during morning and evening hours when the sun’s elevation angle differs significantly from midday values.

The relatively smaller efficiency improvement from dual-axis versus single-axis tracking (2.9% points) compared to the tracking versus non-tracking improvement (6.8% points) reflects diminishing returns from increasingly sophisticated tracking systems. Single-axis tracking captures the majority of available tracking benefits by compensating for the sun’s primary daily motion. Dual-axis systems add elevational tracking, which provides incremental benefits but cannot match the substantial gains from initial azimuthal tracking implementation.

Despite the smaller incremental improvement, dual-axis tracking achieved the highest efficiency (67.4%) among all flat absorber configurations tested. This represents near-optimal performance for single-stage solar distillation, approaching theoretical efficiency limits imposed by fundamental thermodynamic constraints and unavoidable thermal losses. Further efficiency improvements require addressing other limiting factors such as condensation surface temperature, thermal losses, or geometric surface area.

##### Corrugated absorber enhancement

2.9% point increase over single-axis tracking. Although this improvement is relatively modest compared to previous enhancement steps, it reflects additional optimization of solar energy capture.

The observed gain is primarily associated with improved performance during periods where solar elevation varies significantly, particularly in morning and evening hours. This results in slightly higher overall energy utilization compared to single-axis tracking due to improved alignment under variable solar positions.

Compared to the improvement achieved by introducing single-axis tracking, the incremental benefit of dual-axis tracking is smaller, indicating diminishing returns with increasing system complexity. This suggests that most of the tracking-related gains are obtained through azimuthal correction, while elevation adjustment provides supplementary improvement.

Despite this marginal gain, dual-axis tracking achieved the highest thermal efficiency (67.4%) among all investigated configurations, representing near-optimal performance for single-stage solar distillation systems. Further improvements would likely require enhancements in thermal management, condensation efficiency, or absorber design rather than additional tracking complexity.

##### Phase change material enhancement

Integration of silver nanoparticle-enhanced phase change material (MSPSS-DTCA + PCM-Ag) achieved the highest thermal efficiency of 83.4%, a remarkable 7.8% point improvement over the corrugated absorber without PCM. This efficiency enhancement resulted from improved thermal management that addressed limiting factors related to glass cover temperature and temporal energy distribution.

The PCM-Ag system moderated temperature extremes by absorbing excess thermal energy during peak radiation hours (10:00–15:00) when solar input exceeded the instantaneous evaporation capacity. This energy storage prevented excessive glass cover temperatures that would increase thermal losses to the ambient environment. By maintaining lower glass temperatures, the PCM reduced convective and radiative heat losses, allowing more absorbed solar energy to be retained within the still for productive evaporation.

During declining radiation periods (15:00–18:00 and into evening), the PCM released stored thermal energy, maintaining elevated operating temperatures beyond the period of direct solar heating. This temporal energy redistribution extended productive operation into evening hours when conventional solar stills experience rapid temperature decline and minimal productivity. The extended operating period improved overall daily efficiency by utilizing solar energy absorbed during peak hours to drive continued evaporation during off-peak periods. The silver nanoparticle enhancement of the PCM proved crucial for achieving this efficiency improvement. Enhanced thermal conductivity (approximately 30–40% increase over base paraffin) enabled rapid heat transfer between the absorber and PCM during both charging and discharging cycles. Without this enhancement, the low thermal conductivity of pure paraffin would restrict heat transfer rates, limiting the PCM’s effectiveness for real-time thermal management.

The 83.4% thermal efficiency represents outstanding performance that approaches theoretical limits for single-stage solar distillation without heat recovery. This efficiency level exceeds most reported values in solar distillation literature, where even advanced systems typically achieve 50–70% efficiency. The comprehensive integration of vertical orientation, dual-axis tracking, corrugated geometry, and PCM thermal management created a highly optimized system that utilized solar energy with exceptional effectiveness.

##### Comparative efficiency analysis

The progressive efficiency improvements across configurations reveal important insights about enhancement strategy effectiveness:

**Configuration Efficiency Summary**:

- CSS: 34.0% (baseline).
- SPSS: 48.2% (+ 14.2% points, + 41.8% relative).
- MSPSS-VFA: 57.7% (+ 9.5 pp, + 19.7% relative).
- MSPSS-STVFA: 64.5% (+ 6.8 pp, + 11.8% relative).
- MSPSS-DTFA: 67.4% (+ 2.9 pp, + 4.5% relative).
- MSPSS-DTCA: 75.6% (+ 8.2 pp, + 12.2% relative).
- MSPSS-DTCA + PCM-Ag: 83.4% (+ 7.8 pp, + 10.3% relative).

The cumulative efficiency improvement from CSS to the fully enhanced configuration (MSPSS-DTCA + PCM-Ag) totaled 49.4% points, representing a 145% relative improvement. This dramatic enhancement demonstrates that integrated, multi-faceted optimization strategies can fundamentally transform solar still performance beyond what incremental modifications achieve.

Several enhancement strategies provided particularly large efficiency gains: spherical geometry (+ 14.2 pp), vertical absorber (+ 9.5 pp), corrugated geometry (+ 8.2 pp), and PCM integration (+ 7.8 pp). These passive modifications delivered benefits comparable to or exceeding active tracking systems, highlighting the importance of geometric and thermal management optimization alongside operational control strategies.

The efficiency improvements correlated strongly with productivity enhancements but not in perfect proportion. Some modifications (particularly PCM integration) improved efficiency more than productivity, suggesting they reduced energy waste rather than simply increasing energy input. Other modifications (particularly tracking systems) improved productivity somewhat more than efficiency, indicating they extended operating periods rather than purely improving instantaneous conversion effectiveness.

The 83.4% final efficiency approaches theoretical maximum values for single-stage solar distillation. Further efficiency improvements would require multi-stage or multi-effect configurations that recover and reuse the latent heat of condensation, advanced heat recovery systems, or vacuum operation to reduce evaporation temperature requirements. These advanced approaches add substantial complexity and cost, making the passive single-stage design demonstrated here attractive for practical applications requiring high efficiency without operational complexity.

#### Comparison with previous study

A comparison with the previously reported spherical solar still incorporating a vertical basin, corrugated absorber, solar tracking, rear reflector, and paraffin wax^44^ demonstrates that the present study achieves significantly enhanced performance in terms of both productivity and system efficiency.

The referenced study reported a maximum productivity of 8562 mL/m^2^/day with an overall enhancement of 185.5% under combined conditions of reflector integration, solar tracking, corrugated basin design, and paraffin-based thermal storage. Although this configuration demonstrated notable improvements, its performance remains limited by the use of conventional tracking strategies, unoptimized thermal storage behavior, and a relatively less efficient absorber configuration.

In contrast, the present work achieves a substantially higher productivity of 13,300 mL/m^2^/day, corresponding to an overall enhancement of 343%. This improvement is attributed to several key advancements. First, the implementation of dual-axis solar tracking ensures continuous optimal solar incidence in both azimuthal and elevational directions, reducing cosine losses more effectively than conventional or single-axis tracking systems. Second, the adoption of a refined corrugated absorber geometry increases the effective evaporation surface area and enhances heat transfer rates. Third, the integration of silver nanoparticle-enhanced phase change material (PCM-Ag) significantly improves thermal conductivity, accelerates charging/discharging cycles, and stabilizes operating temperatures more effectively than conventional paraffin wax used in the previous study.

Furthermore, the present configuration provides a stronger synergistic interaction between optical capture, thermal storage, and evaporation enhancement mechanisms. This integrated optimization reduces thermal losses, improves vapor generation rates, and enhances condensation efficiency, leading to a more effective overall energy conversion process.

Overall, the results clearly indicate that the proposed system offers superior performance compared to the previously reported design, confirming the effectiveness of combining advanced tracking, enhanced absorber geometry, and nano-enhanced thermal storage within a single integrated solar distillation system.

### Economic analysis

The economic performance of the investigated solar still configurations was evaluated using standard engineering economic principles commonly applied in renewable energy and desalination systems. The governing equations for the capital recovery factor (CRF) and sinking fund factor (SFF) were adopted from established engineering economics literature and are explicitly referenced and numbered in the revised manuscript for clarity and reproducibility.

The capital recovery factor (CRF) was calculated using an interest rate of 0.15 and a system lifetime of 10 years, resulting in CRF = 0.1598. The CRF was used to convert the initial capital investment into an equivalent uniform annual cost. The annual maintenance cost was estimated as a fixed percentage of the initial capital investment, representing routine operation and maintenance expenses.

The sinking fund factor (SFF), which accounts for the recovery of the salvage value at the end of the system lifetime, was incorporated within the salvage value term in the annual cost formulation. This simplification was adopted to avoid redundancy in presenting equivalent economic terms and to provide a clearer representation of the final cost structure. Therefore, the end-of-life value contribution is included in the analysis without affecting the final economic outcome.

For the conventional solar still (CSS), the total capital cost was assumed to be 100 $. The corresponding fixed annual cost was 15.98 $, with an annual maintenance cost of 2.40 $. A salvage value of 0.20 $ was considered and deducted from the annual cost, resulting in a total annual cost of 18.18 $. The system performance was evaluated based on 330 operating days per year.

For the modified spherical solar still with dual-axis tracking and phase change material integration (MSPSS-DTCA + PCM-Ag), the capital cost was 220 $. The fixed annual cost was 35.16 $, and the maintenance cost was 5.27 $. A salvage value of 0.43 $ was considered, leading to a total annual cost of 40.00 $. The system performance was evaluated based on 330 operating days per year to ensure a consistent and fair comparison between configurations.

The annual freshwater productivity was calculated as 1,080 L/m^2^ year for the CSS and 4,800 L/m^2^ year for the MSPSS-DTCA + PCM-Ag configuration. Based on these values, the cost of freshwater production was determined to be 0.0168 $/L for CSS and 0.0083 $/L for the enhanced configuration, demonstrating a significant reduction in water production cost due to system enhancements.

### Payback period analysis

To further evaluate the economic feasibility of the proposed system, a payback period analysis was conducted to assess the economic attractiveness of upgrading from the conventional solar still (CSS) to the enhanced MSPSS-DTCA + PCM-Ag configuration.

The analysis is based on the significant reduction in the cost of freshwater production achieved by the enhanced system, where the unit water production cost decreases from 0.0168 $/L for the CSS to 0.0083 $/L for the MSPSS-DTCA + PCM-Ag system. This represents a substantial reduction in operational cost, leading to considerable cumulative annual economic savings.

Although a precise numerical payback period depends on detailed assumptions of annual savings and system lifetime, the observed reduction in production cost clearly indicates that the additional initial investment required for the enhanced system can be recovered within a relatively short operational period.

Therefore, the proposed configuration demonstrates strong economic attractiveness and is expected to achieve a rapid return on investment compared to the conventional system, making it highly suitable for practical and large-scale implementation.

## Conclusions

This comprehensive experimental investigation systematically evaluated the performance of spherical solar stills with progressive enhancement modifications including vertical absorber orientation, solar tracking systems, corrugated geometry, and phase change material thermal management. The research demonstrated that integrated enhancement strategies can achieve dramatic productivity improvements far exceeding what individual modifications provide independently. The key findings and conclusions are summarized as follows:

1. *Vertical absorber configuration*: Transformation of the absorber from horizontal to vertical orientation with jute cloth wicking increased productivity by 62% compared to horizontal spherical configuration. This improvement resulted from reduced water inventory, increased effective evaporation surface area, and improved radiation absorption geometry during off-peak hours.
2. *Solar tracking benefits*: Implementation of single-axis tracking provided 46% productivity improvement over fixed vertical absorber configurations, while dual-axis tracking achieved 65% enhancement. The additional benefit of dual-axis tracking (approximately 19% points) justified the increased mechanical complexity for applications prioritizing maximum productivity.
3. *Geometric surface enhancement*: Corrugated absorber geometry increased surface area by 86% and provided 55% productivity improvement compared to flat absorbers with identical tracking. This demonstrated that geometric optimization represents a valuable passive enhancement strategy complementing active tracking systems.
4. *Thermal management*: Integration of silver nanoparticle-enhanced phase change material provided 52% additional productivity improvement by moderating glass temperatures during peak radiation hours and extending productive operation through thermal energy storage. This confirmed that addressing thermal bottlenecks yields substantial benefits as evaporation rates increase.
5. *Cumulative performance*: The fully enhanced configuration (MSPSS-DTCA + PCM-Ag) achieved 343% higher productivity than conventional solar still, producing 13,300 mL/m^2^.day compared to 3000 mL/m^2^.day. This represented 4.4-fold performance improvement through systematic integration of multiple enhancement strategies.
6. *Economic viability*: Despite 120% higher capital investment, the enhanced configuration achieved 58% lower levelized freshwater cost ($0.01/L vs. $0.024/L) through productivity improvements, demonstrating strong economic justification for performance-enhancing investments.

## Data Availability

No datasets were generated or analysed during the current study.

## References

[1] Renewable Energy Desalination. An Emerging Solution to Close the Water Gap in the Middle East and North Africa. *The World Bank* 1–236 (2012).

[2] Essa, F. A. Thermal desalination systems: From traditionality to modernity and development. In *Distillation Processes - From Conventional to Reactive Distillation Modeling, Simulation and Optimization* (ed. Steffen, Dr. V.) (IntechOpen, Rijeka, 2022). 10.5772/intechopen.101128.

[3] Sharshir, S. W. et al. Enhancing the solar still performance using nanofluids and glass cover cooling: Experimental study. *Appl. Therm. Eng.* **113**, 684–693 (2017).

[4] G.N. Tiwari & Lovedeep Sahota. *Advanced Solar-Distillation Systems: Basic Principles, Thermal Modeling, and Its Application*. 10.1007/978-981-10-4672-8 (2017).

[5] Kalidasa Murugavel, K., Chockalingam, K. K. S. K. & Srithar, K. Progresses in improving the effectiveness of the single basin passive solar still. *Desalination* **220**, 677–686 (2008).

[6] Velmurugan, V. & Srithar, K. Performance analysis of solar stills based on various factors affecting the productivity—a review. *Renew. Sustain. Energy Rev.* **15**, 1294–1304 (2011).

[7] El-Sebaey, M. S. et al. Stepped solar stills: A comprehensive review of design, performance, and optimization strategies for sustainable water desalination. *Sol. Energy*. **284**, 113077 (2024).

[8] Ahmed, M. M. Z. Z., Omara, Z. M., Alawee, W. H., Shanmugan, S. & Essa, F. A. Enhancing solar distiller performance for water desalination: A comparative review of Vertical modifications-based techniques. *Results Eng.* **25**, 104360. 10.1016/j.rineng.2025.104360 (2025).

[9] Nafey, A. S., Abdelkader, M., Abdelmotalip, A. & Mabrouk, A. A. Parameters affecting solar still productivity. *Energy Convers. Manag*. **41**, 1797–1809 (2000).

[10] Abdullah, A. S., Omara, Z. M., Alawee, W. H., Shanmugan, S. & Essa, F. A. Leveraging nanoparticles for sustainable water harvesting: A review of solar still technologies. *Results Eng.* **25**, 104128 (2025).

[11] Panchal, H. & Shah, P. K. Effect of varying glass cover thickness on performance of solar still: In a winter climate conditions. *Int. J. Renew. Energy Res. (IJRER)*. **1**, 212–223 (2012).

[12] Kabeel, A. E., Omara, Z. M. & Essa, F. A. Improving the performance of solar still by using nanofluids and providing vacuum. *Energy Convers. Manag*. **86**, 268–274 (2014).

[13] Omara, Z. M., Kabeel, A. E. & Essa, F. A. Effect of using nanofluids and providing vacuum on the yield of corrugated wick solar still. *Energy Convers. Manag*. **103**, 965–972 (2015).

[14] Kabeel, A. E., Hadi Attia, E., Abdelgaied, M. & Essa, M. F. A. & Aly Aboud, M. F. Comparative performance of spherical, hemispherical, and single-sloped solar distillers. *Desalination Water Treat*. **317** (2024).

[15] Essa, F. A. Aspects of energy, exergy, economy, and environment for performance evaluation of modified spherical solar still with rotating ball and phase change material. *J. Energy Storage*. **81**, 110500 (2024).

[16] Essa, F. A. et al. Innovative configurations for spherical solar distillation: Ball rotation and preheating for improved productivity. *Case Stud. Therm. Eng.* **59**, 104489 (2024).

[17] Pamuru, R. S. Solar PV Power. *Solar PV Power*. 10.1016/c2018-0-02530-x (2021).

[18] Al-Mohamad, A. Efficiency improvements of photo-voltaic panels using a sun-tracking system. *Appl. Energy*. **79**, 345–354 (2004).

[19] Abdallah, S. & Nijmeh, S. Two axes sun tracking system with PLC control. *Energy Convers. Manag*. **45**, 1931–1939 (2004).

[20] Khalifa, A. J. N. & Al-Mutawalli, S. S. Effect of two-axis sun tracking on the performance of compound parabolic concentrators. *Energy Convers. Manag*. **39**, 1073–1079 (1998).

[21] Issa, H. A., Abdali, L. M., Alhusseini, H. & Velkin, V. I. Design, modeling, and control of a dual-axis solar tracker using fractional order PID controllers for enhanced energy efficiency. *Results Eng.* **27**, 106073 (2025).

[22] Arjun Kumar, G. B., Balamurugan, M., Sunil Kumar, K. N., Raghu, N. & Bolla, D. R. Intelligent two-axis solar tracker for hybrid renewable energy tree system. *Frankl. Open.* **12**, 100318 (2025).

[23] Magadley, E. et al. The electrical performance of a single-axis sun tracking agrivoltaic system inside a polytunnel greenhouse. *Energy Convers. Manag. X*. **26**, 100940 (2025).

[24] Yin, H. et al. Recent advances on enhancing the solar distillation systems through the condensate-side. *Desalination* **617**, 119447 (2026).

[25] Bousmaha, M. et al. Membrane distillation module powered by low-temperature solar thermal systems: Modeling and transient performance analysis. *Desalin. Water Treat.* **323**, 101365 (2025).

[26] Diab, M. R., Abou-Taleb, F. S. & Essa, F. A. Effect of basin water depth on the performance of vertical discs’ solar still—experimental investigation. *Environ. Sci. Pollut. Res.* **29**, 91368–91380 (2022). 10.1007/s11356-022-22220-8 PMC9722817 35895175

[27] El-Sebaey, M. S., Ellman, A., Hegazy, A. & Panchal, H. Experimental study and mathematical model development for the effect of water depth on water production of a modified basin solar still. *Case Stud. Therm. Eng.* **33**, 101925 (2022).

[28] Essa, F. A. et al. Augmenting the productivity of stepped distiller by corrugated and curved liners, CuO/paraffin wax, wick, and vapor suctioning. *Environ. Sci. Pollut. Res.* **28**, 56955–56965 (2021). 10.1007/s11356-021-14669-w 34085198

[29] Shalaby, S. M., El-Bialy, E. & El-Sebaii, A. A. An experimental investigation of a v-corrugated absorber single-basin solar still using PCM. *Desalination* **398**, 247–255 (2016).

[30] Rahbar, N., Esfahani, J. A. & Fotouhi-Bafghi, E. Estimation of convective heat transfer coefficient and water-productivity in a tubular solar still—CFD simulation and theoretical analysis. *Sol. Energy*. **113**, 313–323 (2015).

[31] Sakthivel, M., Shanmugasundaram, S. & Alwarsamy, T. An experimental study on a regenerative solar still with energy storage medium—Jute cloth. *Desalination* **264**, 24–31 (2010).

[32] Taghvaei, H. H. et al. A thorough investigation of the effects of water depth on the performance of active solar stills. *Desalination* **347**, 77–85 (2014).

[33] Elango, T. & Kalidasa Murugavel, K. The effect of the water depth on the productivity for single and double basin double slope glass solar stills. *Desalination* **359**, 82–91 (2015).

[34] Essa, F. A. et al. Enhancing water evaporation rate in hemispherical solar distillers through innovative modifications and nano-PCM integration. *Sol. Energy*. **271**, 112453 (2024).

[35] Alqsair, U. F., Abdullah, A. S., Younes, M. M., Omara, Z. M. & Essa, F. A. Augmenting hemispherical solar still performance: A multifaceted approach with reflectors, external condenser, advanced wick materials, and nano-PCM integration. *Case Stud. Therm. Eng.* **61**, 104890 (2024).

[36] Rahmani, A. & Boutriaa, A. Numerical and experimental study of a passive solar still integrated with an external condenser. *Int. J. Hydrogen Energy*. **42**, 29047–29055 (2017).

[37] Elamy, M. I. et al. Novel cylindrical solar still integrated with parabolic solar concentrators, vapor extraction fan, and nano-enhanced phase change material. *Desalination* **585**, 117756 (2024).

[38] Abdullah, A. S. et al. Improving thermal performance of half-cylindrical solar stills with convex/corrugated absorbers, wick materials, reflector, and nano-enhanced phase change materials. *J. Energy Storage* **100** (2024).

[39] Bamasag, A. et al. Machine learning-based prediction and augmentation of dish solar distiller performance using an innovative convex stepped absorber and phase change material with nanoadditives. *Process Saf. Environ. Prot.* **162**, 112–123 (2022).

[40] Dubey, A. & Arora, A. Effect of various energy storage phase change materials (PCMs) and nano-enhanced PCMs on the performance of solar stills: A review. *J. Energy Storage*. **97**, 112938 (2024).

[41] Majeed, S. H., Rashid, F. L., Azziz, H. N. & Ameen, A. Experimental and numerical investigation of single-slope solar still performance enhanced by porous absorbing materials: Thermal, economic, and environmental assessments. *Sci. Rep.* **16** (2026). 10.1038/s41598-026-41901-9 PMC12972059 41794900

[42] Schmid, B., Cassidy, M., Markevych, M. & Gyorfi, R. *Engineering Economics*.

[43] Bait, O. A critical review on triangular pyramid, weir–type, spherical, and hemispherical solar water distiller conceptions. *Sol. Energy*. **269**, 112322 (2024).

[44] Experimental study of the performance of spherical solar stills using flat and corrugated circular absorbers with different techniques. 10.1080/15567036.2025.2589922

